# MHD Flow and Heat Transfer between Coaxial Rotating Stretchable Disks in a Thermally Stratified Medium

**DOI:** 10.1371/journal.pone.0155899

**Published:** 2016-05-24

**Authors:** Tasawar Hayat, Sumaira Qayyum, Maria Imtiaz, Ahmed Alsaedi

**Affiliations:** 1 Department of Mathematics, Quaid-I-Azam University 45320, Islamabad, Pakistan; 2 Nonlinear Analysis and Applied Mathematics (NAAM) Research Group, Department of Mathematics, Faculty of Science, King Abdulaziz University, Jeddah, Saudi Arabia; Tianjin University, CHINA

## Abstract

This paper investigates the unsteady MHD flow of viscous fluid between two parallel rotating disks. Fluid fills the porous space. Energy equation has been constructed by taking Joule heating, thermal stratification and radiation effects into consideration. We convert system of partial differential equations into system of highly nonlinear ordinary differential equations after employing the suitable transformations. Convergent series solutions are obtained. Behavior of different involved parameters on velocity and temperature profiles is examined graphically. Numerical values of skin friction coefficient and Nusselt number are computed and inspected. It is found that tangential velocity profile is increasing function of rotational parameter. Fluid temperature reduces for increasing values of thermal stratification parameter. At upper disk heat transfer rate enhances for larger values of Eckert and Prandtl numbers.

## 1. Introduction

No doubt the fluid flow by rotating disks analyzed extensively because of its many industrial and engineering applications. Such consideration has relevance in medical equipment, food processing technology, air cleaning machine, electric power generating system, aero dynamical engineering, food processing machines and gas turbines. Initial work on rotating disk is presented by Karman [1]. He formed ordinary differential equations from Navier-Stokes equations by using special type of transformations called Von Karman transformations. Later on various researchers used these transformations to examine different physical phenomenon. Cochran [2] used numerical integration for more reliable solution of fluid flow due to a rotating disk. Stewartson [3] examined the fluid flow between two rotating disks. Later on Chapple and Stokes [4] and Mellor et al. [5] studied the fluid flow when one disk is rotating and other stationary. Arora and Stokes [6] analyzed the heat transfer between two rotating disks. MHD flow of fluid between stationary porous disk and solid rotating disk is studied by Kumar et al. [7]. Yan and Soong [8] worked on flow and heat transfer between two rotating porous disks with wall transpiration. Soong et al. [9] analyzed the fluid flow independently between two coaxial rotating disks. Turkyilmazoglu [10] analyzed the rate of heat transfer of the fluid flow over a shrinking surface of rotating disk. The recent advance research in the study of two-phase fluid flow is done by Gao et al. [11–14].

Impact of stratification is very important for heat transfer analysis. Stratification phenomena occur due to variation in temperature or fluid with different densities. Thermal stratification has many applications in reservoirs, oceans, estuaries, salinity stratification in rivers, industrial food, heterogeneous mixtures in atmosphere and manufacturing processes. Zhang et al. [15] examined the turbulent penetration and thermal stratification in a pressurizer surge line with an overall out-surge flow. Flow and heat transfer by an exponentially stretching sheet with thermal stratification effect are analyzed by Mukhopadhyay [16]. Nanofluid flow with effects of double stratification and MHD is discussed by Hayat et al. [17]. Jeffrey fluid flow due to stretching sheet with double stratification effects is also examined by Hayat et al. [18]. Srinivasacharya and Upendar [19]discussed the MHD flow of micropolar fluid with double stratification and free convection.

Study of fluid flow with magnetic field has many applications in physics, chemistry, engineering, metallurgy, polymer industry, power generators, pump, droplet filters, electrostatic filters, reactors cooling, the design of heat exchangers, and accelerators. In such examples rate of heat cooling has vital role to improve the desired characteristics of the final product. Intensity and orientation of magnetic field strongly affect the characteristics of the flow. Heat transfer characteristics of the flow strongly changed when magnetic field is applied because it manipulates the suspended particles of fluid and rearranges their concentration in the fluid. Nanofluid flow with convective conditions and MHD effects is inspected by Hayat et al. [20]. Sheikholeslami et al. [21] studied the flow of Cu-water nanofluid in an inclined half-annulus enclosure with effect of magnetic field on natural convection. MHD flow of nanofluid with mixed convection and slip boundary on a stretching sheet is analyzed by Hsiao [22]. Zhang et al. [23] discussed the nanofluids flow and heat transfer in porous media with MHD and radiation effects. Nanofluid flow past a bidirectional exponentially stretching sheet with MHD effect is examined by Ahmad et al. [24]. Rashidi et al. [25] worked on stream wise transverse MHD flow with heat transfer around a porous obstacle. Sheikholeslami and Ellahi [26] analyzed the simulation of ferrofluid flow for magnetic drug targeting. Hsiao [27] inspected the MHD flow of viscoelastic fluid past a porous wedge with mixed convection. MHD mixed convection flow of viscoelastic fluid over a stretching sheet with Ohmic dissipation is studied by Hsiao [28]. Ganji and Malvandi [29] analyzed the natural convection of nanofluid inside a vertical enclosure in the presence of a uniform magnetic field.

Fluid flow saturating porous medium is quite a natural mechanism just like circulation of H_2_*O* in plants and trees, flow of fluids and solutes in biological tissues and melting and metamorphism of snow. Recently porous medium has gained much interest of researchers because it has many industrial applications like paper pulp drying, gas management in fuel cell, detergent tablets, drying of foods and biological processes such as diffusion, capillarity, dissolution, adsorption, clogging, degradation and shrinkage. Moneim and Hassanin [30] described the MHD flow with a porous medium and oscillatory suction. Ellahi et al. [31] analyzed the MHD peristaltic flow of Jeffrey fluid in a rectangular duct through porous media. Hayat et al. [32] studied the MHD flow of nanofluid over a porous shrinking surface. Yang and Shen [33] studied effects of the porous media distribution on the performance improvement for isothermal chamber. Abad et al. [34] worked for radiation effect in concentrated solar air-heaters filled with a porous medium. Heat transfer in flow of nanofluid in a porous channel with contracting and expanding walls is inspected by Hatami et al. [35]. Zhao and Tang [36] applied the Monte Carlo method to simulate the extinction coefficient of silicon carbide porous media. Battacharyya et al. [37] discussed the radiative flow of micropolar fluid and heat transfer due to porous shrinking sheet. Sheikholeslami et al. [38] studied the heat and mass transfer of a micropolar fluid in a porous channel.

The present work studies the MHD flow of fluid between two rotating disks. Important aspects of thermal radiation, Joule heating and stratification effects are taken in consideration. Convergent solutions are obtained by homotopy analysis method [39–42]. Impact of dimensionless parameters on the velocity, temperature, skin friction coefficient and Nusselt number are examined through plots and tabular values.

## 2. Problem Formulation

**Fig 1:**
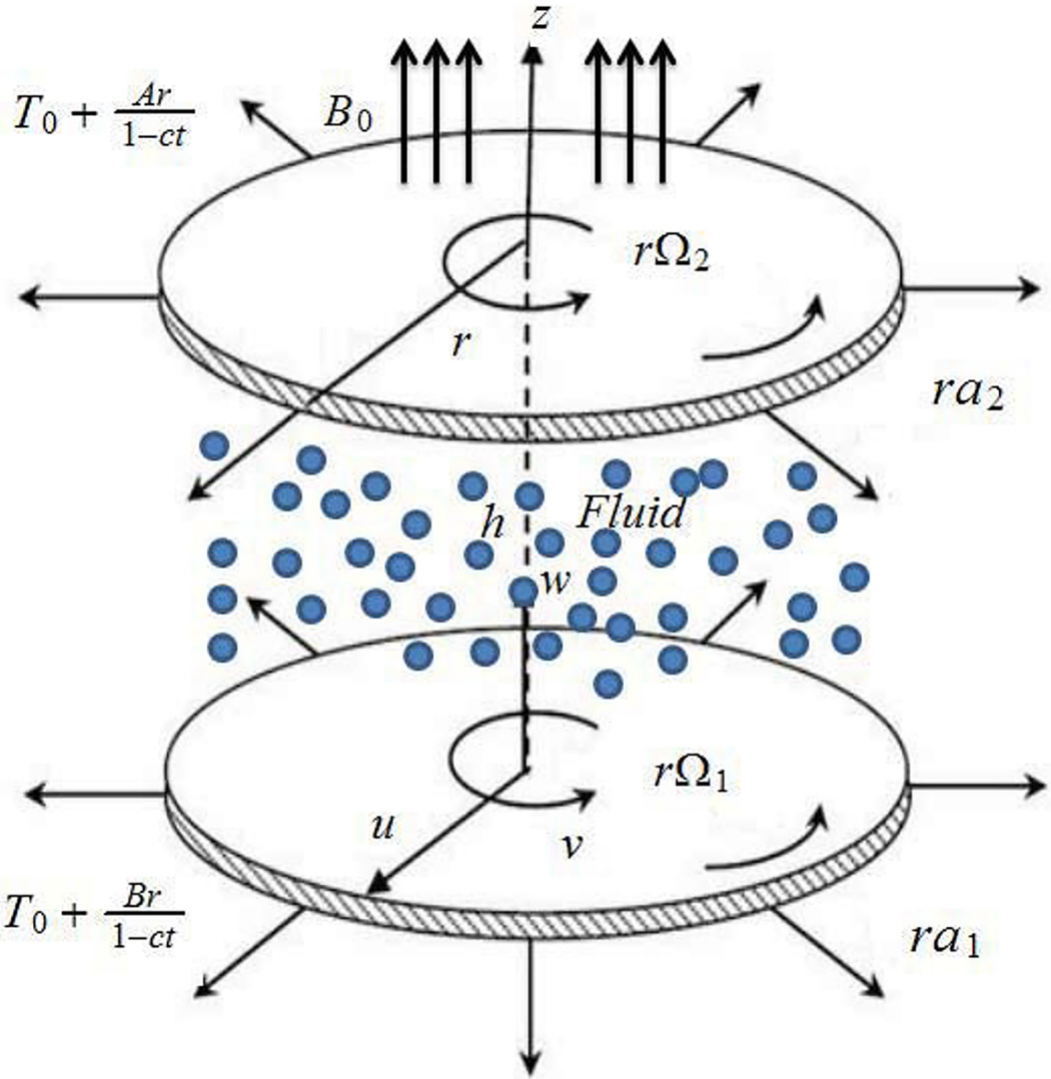
Flow geometry.

Consider an axisymmetric unsteady flow of viscous fluid between two continuously stretching disks. Lower disk is located at *z* = 0 while upper disk is at constant distance *h* apart. Disks are rotating and stretching in axial and radial directions respectively. Disks are rotating with different angular velocities Ω_1_ and Ω_2_ Further the disks are stretching with different rates *a*_1_ and *a*_2_ Lower disk is maintained at temperature $T_{1}(r)=T_{0}+\frac{Ar}{1-ct}$ while temperature at upper disk is $T_{2}(r)=T_{0}+\frac{Br}{1-ct}$ in a thermally stratified medium (see Fig 1).

Fluid filling the porous space with permeability *K*_0_ is also taken in consideration. Magnetic field of strength *B*_0_ is applied parallel to the *z*−axis. Energy equation is constructed in the presence of thermal radiation and Joule heating. The governing equations are

$$\frac{\partial u}{\partial r}+\frac{u}{r}+\frac{\partial w}{\partial z}=0,$$ (1)

$$\frac{\partial u}{\partial t}+u\frac{\partial u}{\partial r}+w\frac{\partial u}{\partial z}-\frac{v^{2}}{r}=-\frac{1}{\rho }\frac{\partial p}{\partial r}+\nu \left(\frac{\partial^{2}u}{\partial r^{2}}+\frac{1}{r}\frac{\partial u}{\partial r}+\frac{\partial^{2}u}{\partial z^{2}}-\frac{u}{r^{2}}\right)-\frac{\sigma }{\rho }B_{0}^{2}u-\frac{\nu }{K_{0}}u,$$ (2)

$$\frac{\partial v}{\partial t}+u\frac{\partial v}{\partial r}+w\frac{\partial v}{\partial z}+\frac{uv}{r}=\nu \left(\frac{\partial^{2}v}{\partial r^{2}}+\frac{1}{r}\frac{\partial v}{\partial r}+\frac{\partial^{2}v}{\partial z^{2}}-\frac{v}{r^{2}}\right)-\frac{\sigma }{\rho }B_{0}^{2}v-\frac{\nu }{K_{0}}v,$$ (3)

$$\frac{\partial w}{\partial t}+u\frac{\partial w}{\partial r}+w\frac{\partial w}{\partial z}=-\frac{1}{\rho }\frac{\partial p}{\partial z}+\nu \left(\frac{\partial^{2}w}{\partial r^{2}}+\frac{1}{r}\frac{\partial w}{\partial r}+\frac{\partial^{2}w}{\partial z^{2}}\right)-\frac{\nu }{K_{0}}w,$$ (4)

$$(\rho c_{p})\left(\frac{\partial T}{\partial t}+u\frac{\partial T}{\partial r}+w\frac{\partial T}{\partial z}\right)=\left(k+\frac{16\sigma^{*}T_{2}^{3}}{3k^{*}}\right)\;\left(\frac{1}{r}\frac{\partial T}{\partial r}+\frac{\partial^{2}T}{\partial r^{2}}+\frac{\partial^{2}T}{\partial z^{2}}\right)+\sigma B_{0}^{2}(u^{2}+v^{2}),$$ (5)

with boundary conditions

u=ra11−ct,  v=rΩ11−ct,  w=0,  T=T1(r)=T0+Ar1−ct at  z=0,u=ra21−ct,  v=rΩ21−ct,  w=0,  T=T2(r)=T0+Br1−ct at  z=h, (6)

where *p* denotes pressure, *ρ* denotes density, *ν* is kinematic viscosity, *σ* is electrical conductivity, *c*_*p*_ is specific heat, *k* is thermal conductivity, σ* is Stefan-Boltzman constant and k* denotes mean absorption coefficient, *T*_0_ is reference temperature, *A* and *B* are dimensional constants with dimension *KL*^−1^, *c* is positive constant with dimension (*Time*)^−1^ and *T* is temperature of fluid.

By using the Von Karman transformations [1]

u=rΩ11−ctf′(ξ),  v=rΩ11−ctg(ξ),  w=−2hΩ11−ctf(ξ),  θ=T−T2T1−T0, p=ρΩ1ν(1−ct)2(P(ξ)+12r2h2ε),  ξ=zh1−ct, (7)

the continuity equation is satisfied identically and Eqs 2–6 take the following forms:

$$f^{\prime \prime \prime }+\text{Re}(2ff^{\prime \prime }+g^{2}-f^{\prime^{2}}-\frac{1}{2}A_{1}\xi f^{\prime \prime }-A_{1}f^{\prime }-Mf^{\prime }-\frac{1}{\beta }f^{\prime })-\varepsilon =0,$$ (8)

$$\text{Re}(2f^{\prime }g-2fg^{\prime }+A_{1}g+\frac{A_{1}\xi }{2}g^{\prime }+Mg+\frac{1}{\beta }g)-g^{\prime \prime }=0,$$ (9)

$$P^{\prime }=\text{Re}(fA_{1}+\xi f^{\prime }A_{1}-4ff^{\prime }+\frac{2}{\beta }f)-2f^{\prime \prime },$$ (10)

$$\frac{1}{\text{Pr}}\frac{1}{\text{Re}}\theta^{\prime \prime }(1+R)+2f\theta^{\prime }-\frac{\xi A_{1}}{2}\theta^{\prime }+MEc(f^{\prime 2}+g^{2})=0,$$ (11)

f(0)=0, f(1)=0,  f′(0)=γ1,  f′(1)=γ2,  g(0)=1,g(1)=Ω,  θ(0)=1−S,  θ(1)=0,  P(0)=0, (12)

where Re is Reynolds number, Pr is Prandtl number, *M* is Hartman number, *γ*_1_ and *γ*_2_ are scaled stretching parameters, Ω is rotation parameter, *A*_1_ is unsteadiness parameter, *S* is thermal stratification parameter, *Ec* is Eckert number and *R* is radiation parameter. These quantities are represented by

Re=Ω1h2ν,  Pr=ρcpνk,  M=B02σ(1−ct)ρΩ1,  γ1=a1Ω1,γ2=a2Ω1,  Ω=Ω2Ω1,  A1=cΩ1,  β=K0Ω1ν(1−ct),  S=BA,Ec=Ω12r2(1−ct)2(T1−T0)cp,  R=−16σ*T233kk* (13)

To get more simplified form and to remove *ε* we differentiate Eq 8 with respect to *ξ* as follows:

$$f^{iv}+\text{Re}\left(2ff^{\prime \prime \prime }+2gg^{\prime }-\frac{3A_{1}}{2}f^{\prime \prime }-\frac{A_{1}\xi }{2}f^{\prime \prime \prime }-Mf^{\prime \prime }-\frac{1}{\beta }f^{\prime \prime }\right)=0,$$ (14)

and we determine the pressure parameter ∈ from Eq 8 by using boundary conditions in the form

$$\in =f^{\prime \prime \prime }(0)+\text{Re}\left[{\left(g(0)\right)}^{2}-{\left(f^{\prime }(0)\right)}^{2}-\frac{A_{1}\xi }{2}f^{\prime \prime }(0)-A_{1}f^{\prime }(0)-Mf^{\prime }(0)-\frac{1}{\beta }f^{\prime }(0)\right].$$ (15)

Also pressure term can be calculated by integrating Eq 10 with respect to *ξ* and taking limit from 0 to *ξ* we arrive at

$$P=2\left[\text{Re}\left(\frac{A_{1}}{2}\xi f-f^{2}+\frac{1}{\beta }\overset{\xi }{\underset{0}{\int }}fd\xi \right)-f^{\prime }+f^{\prime }(0)\right].$$ (16)

Shear stresses at lower rotating disk in radial and tangential directions are *τ*_*zr*_ and *τ*_*zθ*_

$$\tau_{zr}=\mu {\frac{\partial u}{\partial z}|}_{z=0}=\frac{\mu r\Omega_{1}f^{\prime \prime }(0)}{h\sqrt{1-ct}},\;\tau_{z\theta }=\mu {\frac{\partial v}{\partial z}|}_{z=0}=\frac{\mu r\Omega_{1}g^{\prime }(0)}{h\sqrt{1-ct}}.$$ (17)

Total shear stress is defined as

$$\tau_{w}=\sqrt{\tau_{zr}^{2}+\tau_{z\theta }^{2}}.$$ (18)

Skin friction coefficients *C*_*f*1_ and *C*_*f*2_ at the lower and upper disks are

$$C_{f1}=\frac{{\tau_{w}|}_{z=0}}{\rho {\left(\frac{r\Omega_{1}}{\sqrt{1-ct}}\right)}^{2}}=\frac{1}{\text{R}{\text{e}}_{r}}{\left[{\left(f^{\prime \prime }(0)\right)}^{2}+{\left(g^{\prime }(0)\right)}^{2}\right]}^{1/2},$$ (19)

$$C_{f2}=\frac{{\tau_{w}|}_{z=h}}{\rho {\left(\frac{r\Omega_{1}}{\sqrt{1-ct}}\right)}^{2}}=\frac{1}{\text{R}{\text{e}}_{r}}{\left[{\left(f^{\prime \prime }(1)\right)}^{2}+{\left(g^{\prime }(1)\right)}^{2}\right]}^{1/2},$$ (20)

where ${\text{Re}}_{r}=\frac{r\Omega_{1}h}{\nu \sqrt{1-ct}}$ is the local Reynolds number. Heat transfer rates for lower and upper disks are

$$Nu_{x1}={\frac{hq_{w}\sqrt{1-ct}}{k(T_{1}-T_{0})}|}_{z=0},\;Nu_{x2}={\frac{hq_{w}\sqrt{1-ct}}{k(T_{1}-T_{0})}|}_{z=h},$$ (21)

where wall heat flux *q*_*w*_ is given by

qw|z=0=−k∂T∂z+qr|z=0=−k(T1−T0)h1−ct(1+16σ*T23(T1−T0)3k*k) θ′(0), (22)

qw|z=h=−k∂T∂z+qr|z=h=−k(T1−T0)h1−ct(1+16σ*T23(T1−T0)3k*k) θ′(1),. (23)

The Nusselt numbers for lower and upper disks are as follows:

$$Nu_{x1}=-(1+R)\theta^{\prime }(0),\;Nu_{x2}=-(1+R)\theta^{\prime }(1).$$ (24)

## 3. Homotopic Solutions

### 3.1 Zeroth-order deformation problems

Initial guesses *f*_0_(*ξ*), *g*_0_(*ξ*) and *θ*_0_(*ξ*) and auxiliary linear operators L_*f*_, L_*g*_ and L_*θ*_ are taken as follows:

$$f_{0}(\xi )=\gamma_{1}\xi -2\gamma_{1}\xi^{2}-\gamma_{2}\xi^{2}+\gamma_{1}\xi^{3}+\gamma_{2}\xi^{3},$$ (25)

$$g_{0}(\xi )=1-\xi +\gamma_{3}\xi ,$$ (26)

$$\theta_{0}(\xi )=(1-\xi )(1-S),$$ (27)

$${\text{L}}_{f}=f^{\prime \prime \prime \prime },\;{\text{ L}}_{g}=g^{\prime \prime },\;{\text{ L}}_{\theta }=\theta^{\prime \prime },$$ (28)

with

$${\text{L}}_{f}\left[c_{1}+c_{2}\xi +c_{3}\xi^{2}+c_{4}\xi^{3}\right]=0,$$ (29)

$${\text{L}}_{g}[c_{5}+c_{6}\xi ]=0,$$ (30)

$${\text{L}}_{\theta }\left[c_{7}+c_{8}\xi \right]=0,$$ (31)

where *c*_*i*_(*i* = 1−8) are the constants.

Denoting *q*∈[0,1] as the embedding parameter and *ħ*_*f*_, *ħ*_*g*_ and *ħ*_*θ*_ the non-zero auxiliary parameters then the zeroth order deformation problems are

$$(1-q){\text{L}}_{f}\left[F(\xi ;\;q)-f_{0}(\xi )\right]=q\hbar_{f}N_{f}[F(\xi ;\;q),\;G(\xi ;\;q)],$$ (32)

$$(1-q){\text{L}}_{g}\left[G(\xi ;\;q)-g_{0}(\xi )\right]=q\hbar_{g}N_{g}[G(\xi ;\;q),\;F(\xi ;\;q)],$$ (33)

$$(1-q){\text{L}}_{\theta }\left[\hat{\theta }(\xi ;\;q)-\theta_{0}(\xi )\right]=q\hbar_{\theta }N_{\theta }[\hat{\theta }(\xi ;\;q),\;F(\xi ;\;q),\;G(\xi ;\;q)],$$ (34)

$$F(0;\;q)=0,\;F(1;\;q)=0,\;F^{\prime }(0;\;q)=\gamma_{1},\;F^{\prime }(1;\;q)=\gamma_{2},$$ (35)

G(0; q)=1,  G(1; q)=Ω, (36)

$$\hat{\theta }(0;\;q)=1-S,\;\hat{\theta }(1;\;q)=0,$$ (37)

where nonlinear differential operators $N_{f},$, $N_{g}$ and $N_{\theta }$ are

Nf[F(ξ; q), G(ξ; q)]=∂4F(ξ; q)∂ξ4+Re[2G(ξ; q)∂G(ξ; q)∂ξ+2F(ξ; q)∂3F(ξ; q)∂ξ3−3A12∂2F(ξ; q)∂ξ2−A1ξ2∂3F(ξ; q)∂ξ3−M∂2F(ξ; q)∂ξ2−1β∂2F(ξ; q)∂ξ2], (38)

Ng[G(ξ; q), F(ξ; q)]=Re(2∂F(ξ; q)∂ξG(ξ; q)−2F(ξ; q)∂G(ξ; q)∂ξ+A1G(ξ; q) +A1ξ2∂G(ξ; q)∂ξ+MG(ξ; q)+1βG(ξ; q)−∂2G(ξ; q)∂ξ2), (39)

Nθ[θ^(η; q), F(η; q), G(ξ; q)]=1Pr1Re∂2θ^(ξ; q)∂ξ2(1+R)+2F(ξ; q)∂θ^(ξ; q)∂ξ−ξA12∂θ^(ξ; q)∂ξ+MEc[(∂F(ξ; q)∂ξ)2+(G(ξ; q))2)]. (40)

### 3.2 m^*th*^ order deformation problems

The m^*th*^ order deformation problems are

$${\text{L}}_{f}\left[f_{m}(\xi )-\chi_{m}f_{m-1}(\xi )\right]=\hbar_{f}R_{f,\;m}(\xi ),$$ (41)

$${\text{L}}_{g}\left[g_{m}(\xi )-\chi_{m}g_{m-1}(\xi )\right]=\hbar_{g}R_{g,\;m}(\xi ),$$ (42)

$${\text{L}}_{\theta }\left[\theta_{m}(\xi )-\chi_{m}\theta_{m-1}(\xi )\right]=\hbar_{\theta }R_{\theta ,\;m}(\xi ),$$ (43)

$$f_{m}(0)=\frac{\partial f_{m}(0)}{\partial \xi }=\frac{\partial f_{m}(1)}{\partial \xi }=f_{m}(1)=g_{m}(0)=g_{m}(1)=\theta_{m}(0)=\theta_{m}(1)=0,$$ (44)

where $R_{f,\;m}\left(\xi \right),$ $R_{g,\;m}\left(\xi \right)$ and $R_{\theta ,\;m}\left(\xi \right)$ have the following forms:

$$R_{f,\;m}\left(\xi \right)=f_{m-1}^{iv}+\text{Re}\left(2\overset{m-1}{\underset{k=0}{\sum }}(f_{m-1-k}f_{k}^{\prime \prime \prime }+g_{m-1-k}g_{k}^{\prime })-\frac{3A_{1}}{2}f_{m-1}^{\prime \prime }-\frac{A_{1}\xi }{2}f_{m-1}^{\prime \prime \prime }-Mf_{m-1}^{\prime \prime }-\frac{1}{\beta }f_{m-1}^{\prime \prime }\right),$$ (45)

$$R_{g,\;m}\left(\xi \right)=\text{Re}\left(2\overset{m-1}{\underset{k=0}{\sum }}(f_{m-1-k}^{\prime }g_{k}-f_{m-1-k}g_{k}^{\prime })+A_{1}g_{m-1}+\frac{A_{1}\xi }{2}g_{m-1}^{\prime }+Mg_{m-1}+\frac{1}{\beta }g_{m-1}\right)-g_{m-1}^{\prime \prime },$$ (46)

$$R_{\theta ,\;m}(\eta )=\frac{1}{\text{Pr}}\frac{1}{\text{Re}}\theta_{m-1}^{\prime \prime }(1+R)+2\overset{m-1}{\underset{k=0}{\sum }}f_{m-1-k}\theta_{k}^{\prime }+\frac{\xi A_{1}}{2}\theta_{m-1}^{\prime }+MEc\left[{\left(f_{m-1}^{\prime }\right)}^{2}+{\left(g_{m-1}\right)}^{2}\right],$$ (47)

$$\chi_{m}=\{\begin{array}{c} 0,\;m\leq 1\\ 1,\;m>1 \end{array}.$$ (48)

The general solutions (*f*_*m*_, *g*_*m*_, *θ*_*m*_) comprising the special solutions $\left(f_{m}^{*},\;g_{m}^{*},\;\theta_{m}^{*}\right)$ are

$$f_{m}(\xi )=f_{m}^{*}(\xi )+c_{1}+c_{2}\xi +c_{3}\xi^{2}+c_{4}\xi^{3},$$ (49)

$$g_{m}(\xi )=g_{m}^{*}(\xi )+c_{5}+c_{6}\xi ,$$ (50)

$$\theta_{m}(\xi )=\theta_{m}^{*}(\xi )+c_{7}+c_{8}\xi ,$$ (51)

where *c*_*i*_ (*i* = 1−8) are the involved constants.

## 4. Convergence Analysis

**Fig 2:**
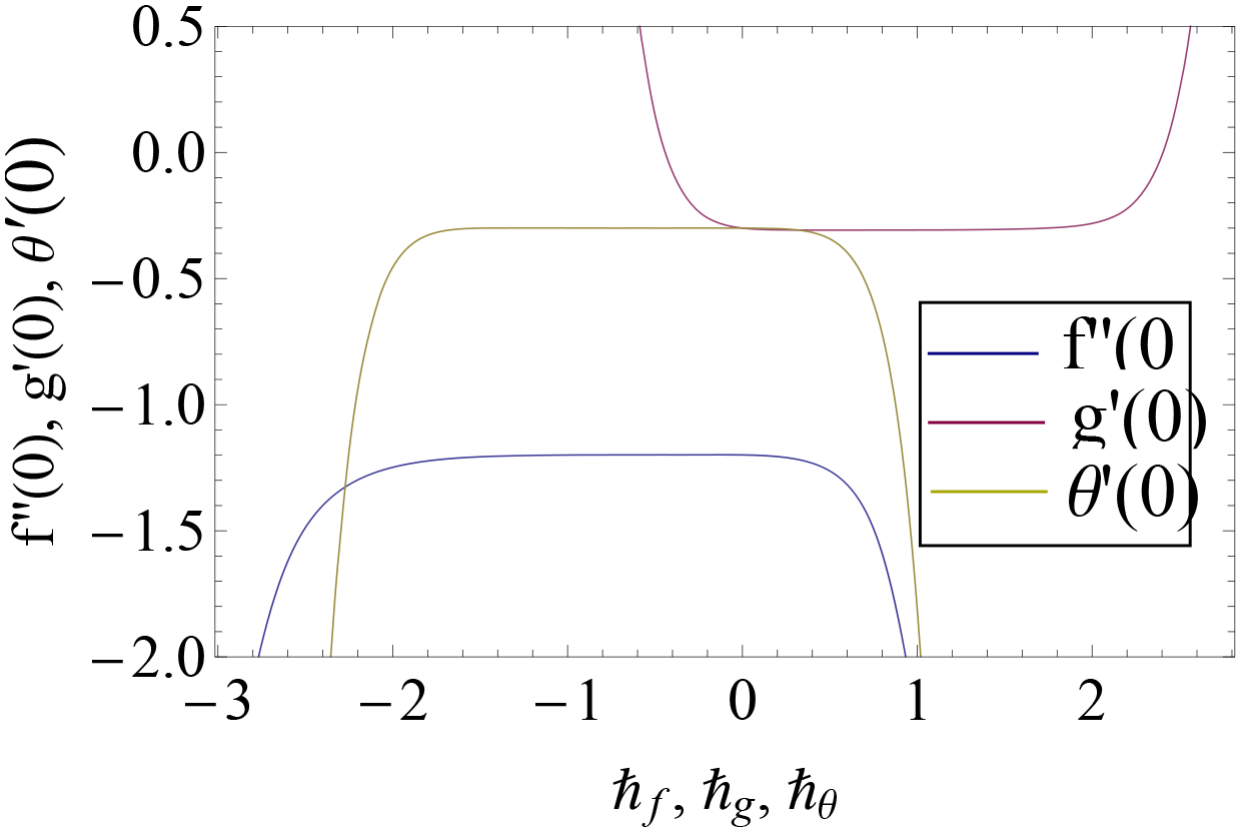
*ħ*− curves for *f*″(0), *g*′(0) and *θ*′(0) when Re = 0.001, *γ*_1_ = 0.1 = *Ec*, Ω = 0.7 = Pr = *S*, *M* = *γ*_2_ = 0.4, *R* = *A*_1_ = 0.5 and *β* = 1.

HAM has great feature to control the convergence region by taking appropriate values of *ħ*_*f*_, *ħ*_*g*_ and *ħ*_*θ*_. We have sketched *ħ*− curves to get valid ranges (see Fig 2). Acceptable values of the auxiliary parameters are −1.25 ≤ *ħ*_*f*_ ≤ −0.01, 0.1 ≤ *ħ*_*g*_ ≤ 1.4 and −1.8 ≤ *ħ*_*θ*_ ≤ −0.05. Series solutions converge in the whole region of *ξ*(0 ≤ *ξ* ≤ ∞) when *ħ*_*f*_ = −0.5, *ħ*_*g*_ = 0.7 and *ħ*_*θ*_ = −0.7.

Table 1 ensures that series solutions of functions *f*″(0), *g*′(0) and *θ*′(0) are convergent up to seven decimal places. We noticed that 11^*th*^, 10^*th*^ and 4^*th*^ order of approximations are appropriate for convergence of *f*″(0), *g*′(0) and *θ*′(0) respectively.

**Table 1:** Convergence of series solutions when Re = 0.001, *γ*_1_ = 0.1 = *Ec*, Ω = 0.7 = Pr = *S*, *M* = *γ*_2_ = 0.4, *R* = *A*_1_ = 0.5 and *β* = 1.

Order of approximation	−*f*″(0)	−*g*′(0)	−*θ*′(0)
1	1.199455	0.3055475	0.2998334
4	1.198978	0.3078518	0.2998416
10	1.198911	0.3079152	0.2998416
11	1.198910	0.3079152	0.2998416
20	1.198910	0.3079152	0.2998416
25	1.198910	0.3079152	0.2998416
30	1.198910	0.3079152	0.2998416
35	1.198910	0.3079152	0.2998416
40	1.198910	0.3079152	0.2998416
45	1.198910	0.3079152	0.2998416
50	1.198910	0.3079152	0.2998416

## 5. Discussion

This section describes the impact of various involved parameters on the velocities, temperature, Nusselt number and skin friction coefficient. For this purpose the graphs and tables are constructed.

### 5.1 Radial and axial velocity profiles

**Fig 3:**
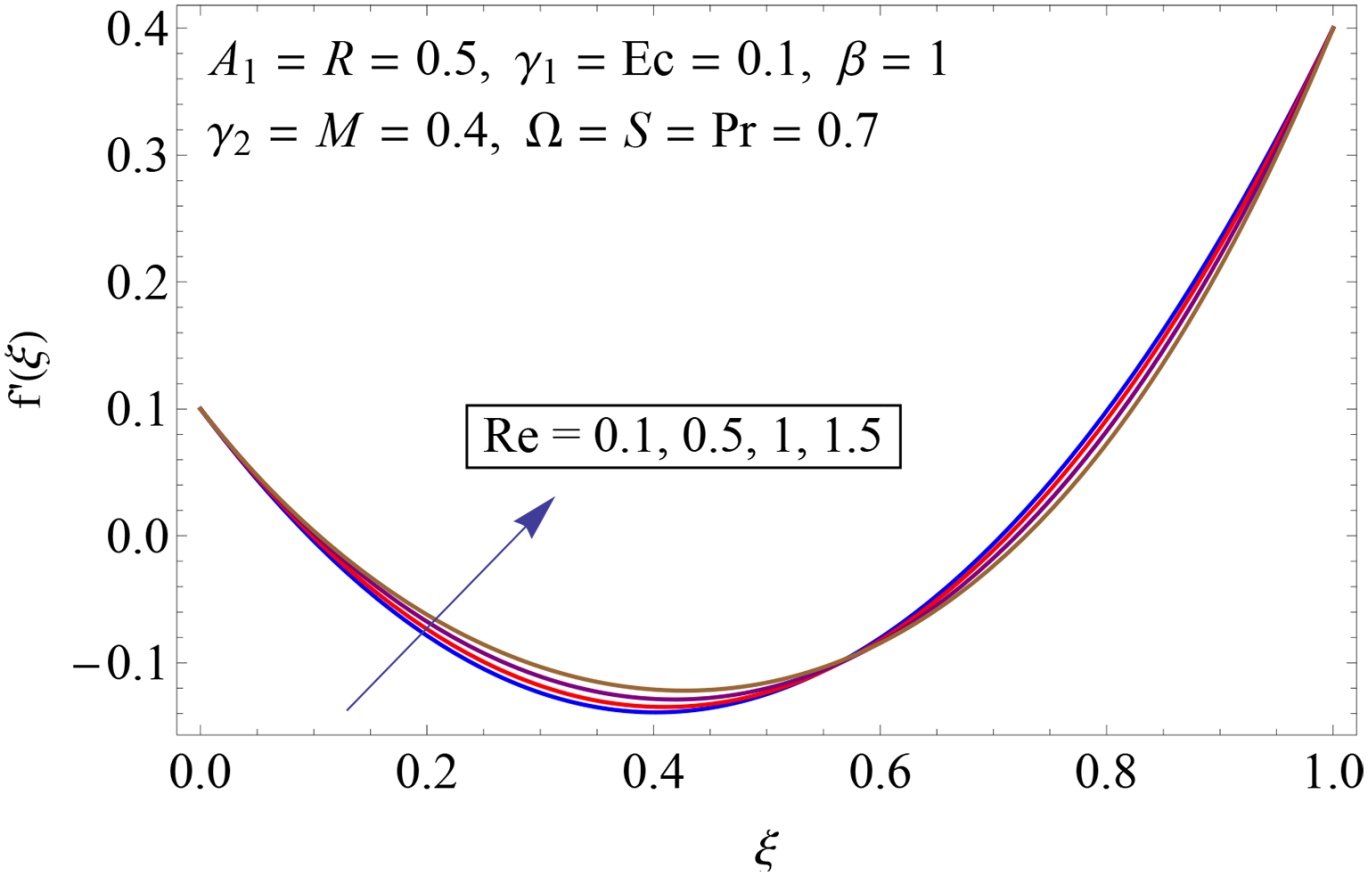
Impact of Re on *f*′(*ξ*).

**Fig 4:**
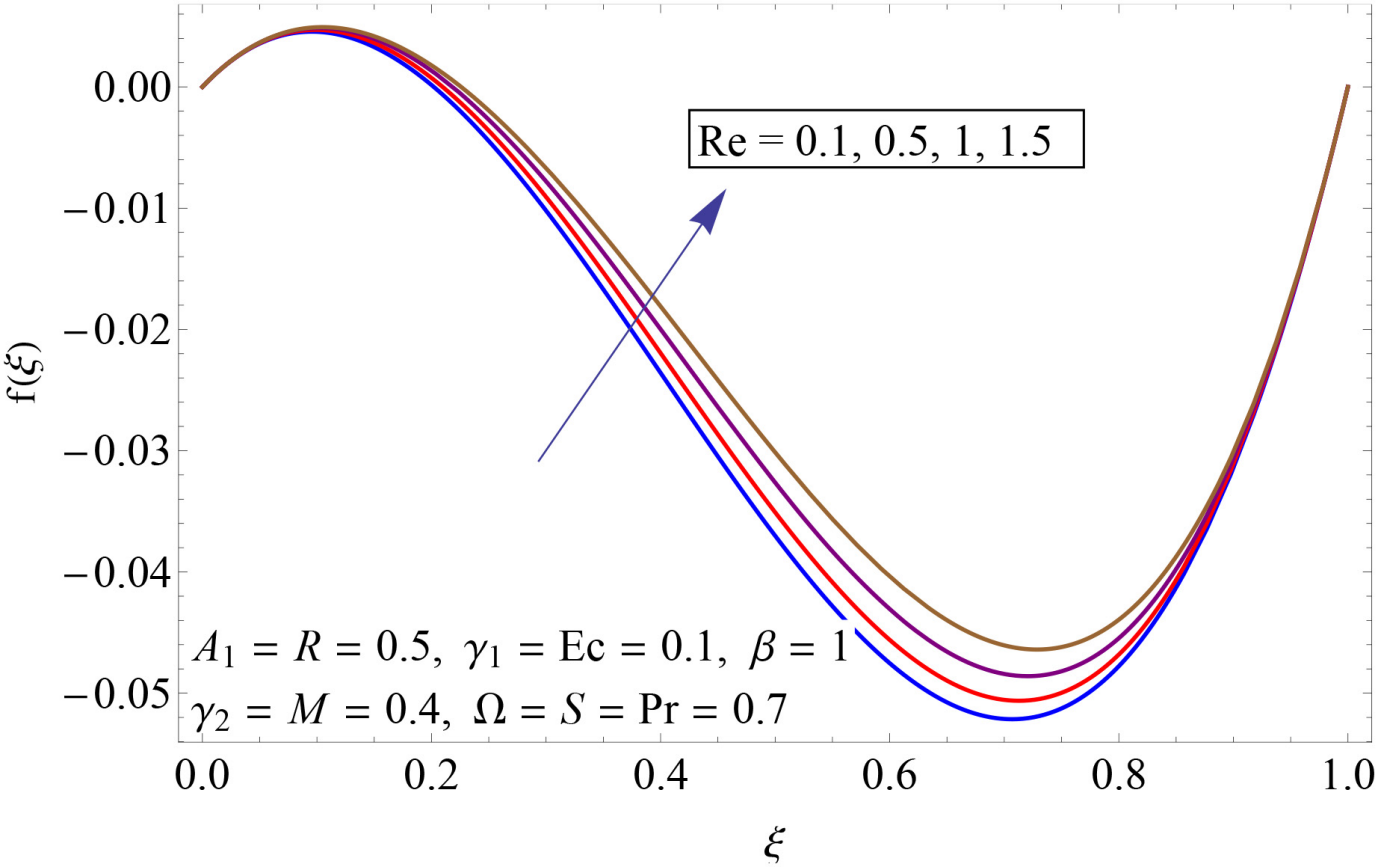
Impact of Re on *f*(*ξ*).

**Fig 5:**
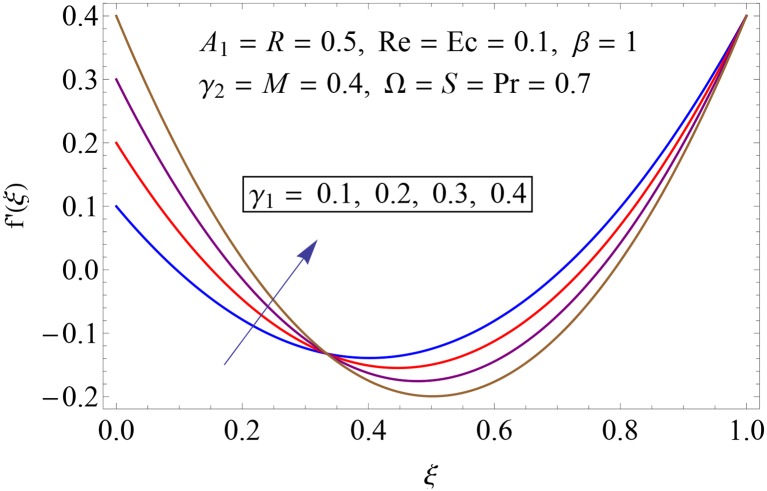
Impact of *γ*_1_ on *f*′(*ξ*).

**Fig 6:**
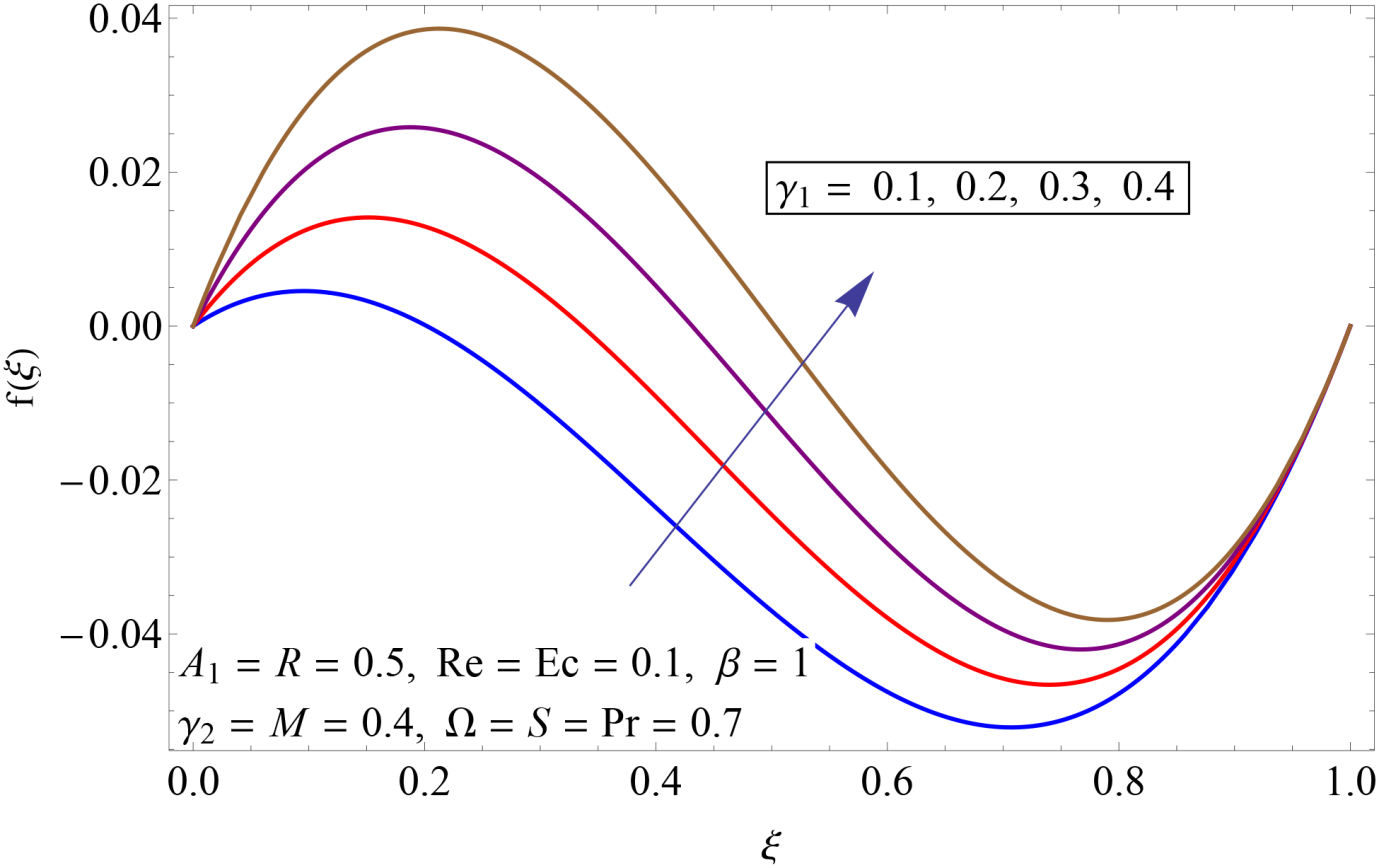
Impact of *γ*_1_ on *f*(*ξ*).

**Fig 7:**
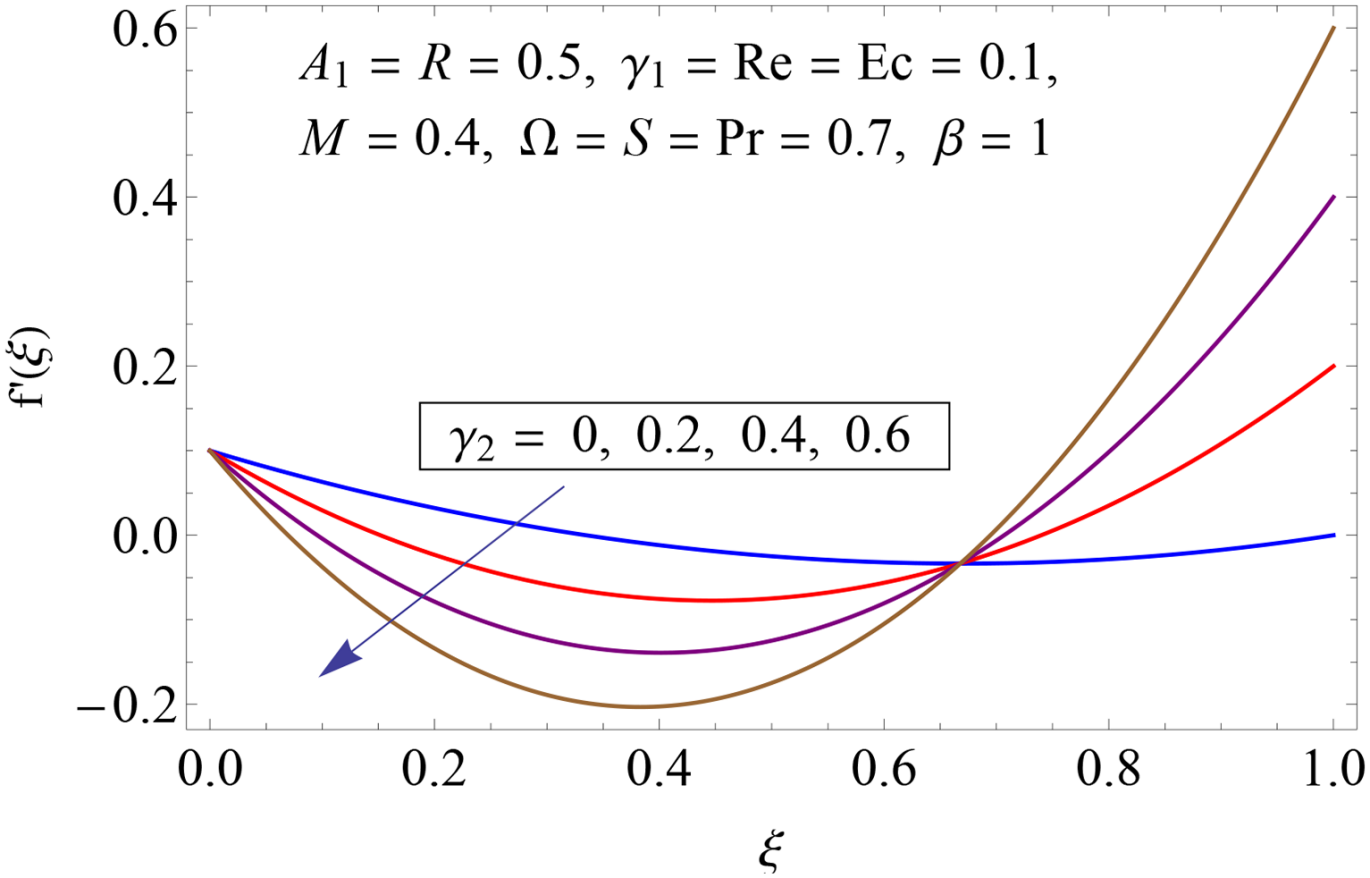
Impact of *γ*_2_ on *f*′(*ξ*).

**Fig 8:**
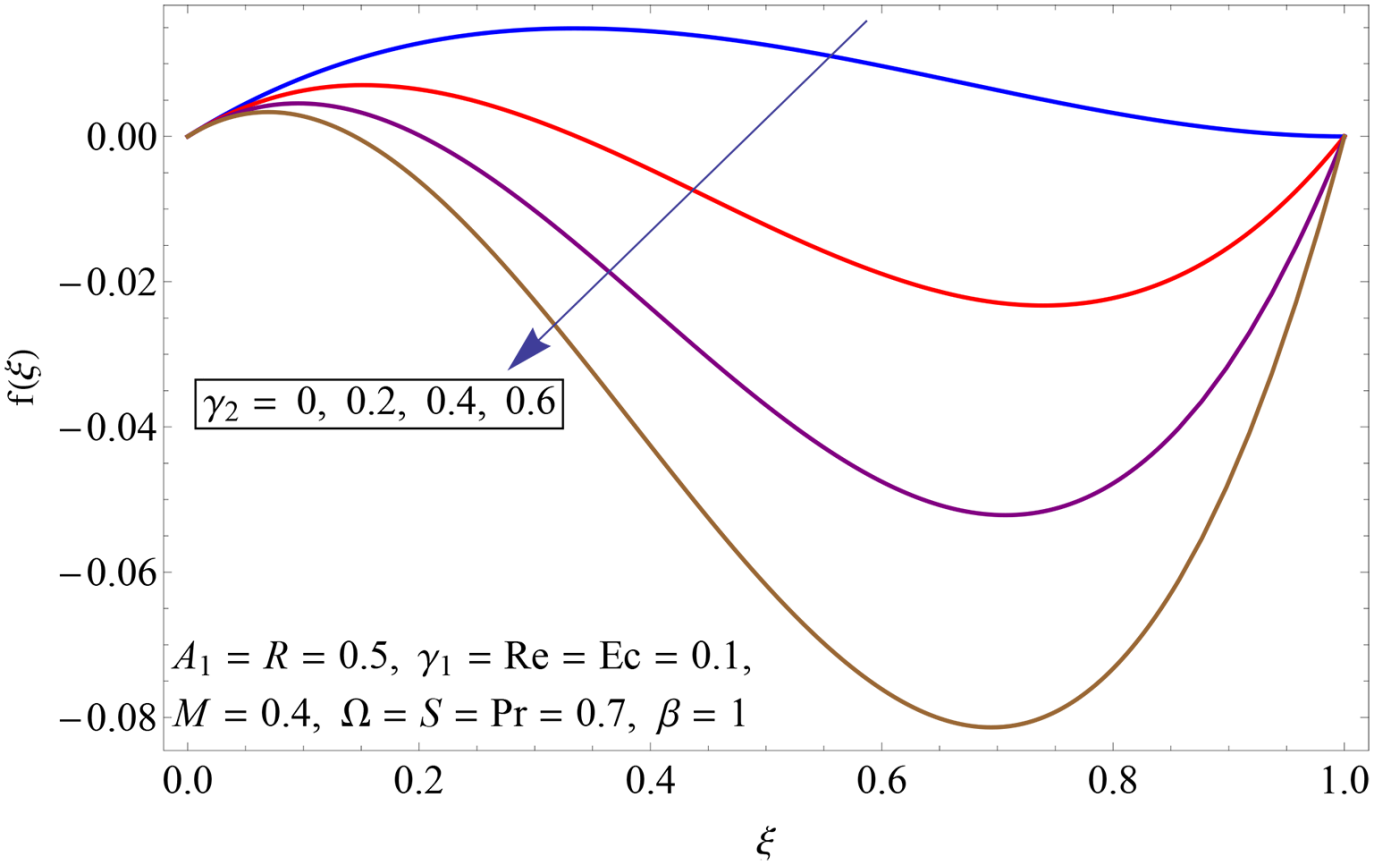
Impact of *γ*_2_ on *f*(*ξ*).

Figs 3–8 represent the behavior of radial *f*′(*ξ*) and axial velocity *f*(*ξ*) profiles for Reynolds number Re and scaled stretching parameters *γ*_1_ and *γ*_2_. Figs 3 and 4 show the impact of Reynolds number Re on radial and axial velocity profiles respectively. Magnitude of radial and axial velocity profiles decreases for increasing values of Re. It is due to the fact that as we increase the values of Reynolds number the inertial force increases which reduce the fluid motion. Upper disk is moving faster than the lower disk so axial velocity has negative values near the lower disk. Figs 5 and 6 show the effect of stretching parameter of lower disk *γ*_1_ on the radial and axial velocity profiles. It is observed that magnitude of radial velocity increases near the lower disk while opposite behavior is noted near upper disk. However axial velocity increases throughout the system. Radial and axial velocity profiles take negative values near the upper disk because stretching at lower disk is increasing. Figs 7 and 8 depict the influence of stretching parameter of upper disk *γ*_2_ on radial and axial velocity of fluid. For larger values of *γ*_2_ the radial velocity of fluid enhances near the upper disk and opposite behavior is noticed at lower disk. However the result of axial velocity is reverse.

### 5.2 Tangential velocity profile

**Fig 9:**
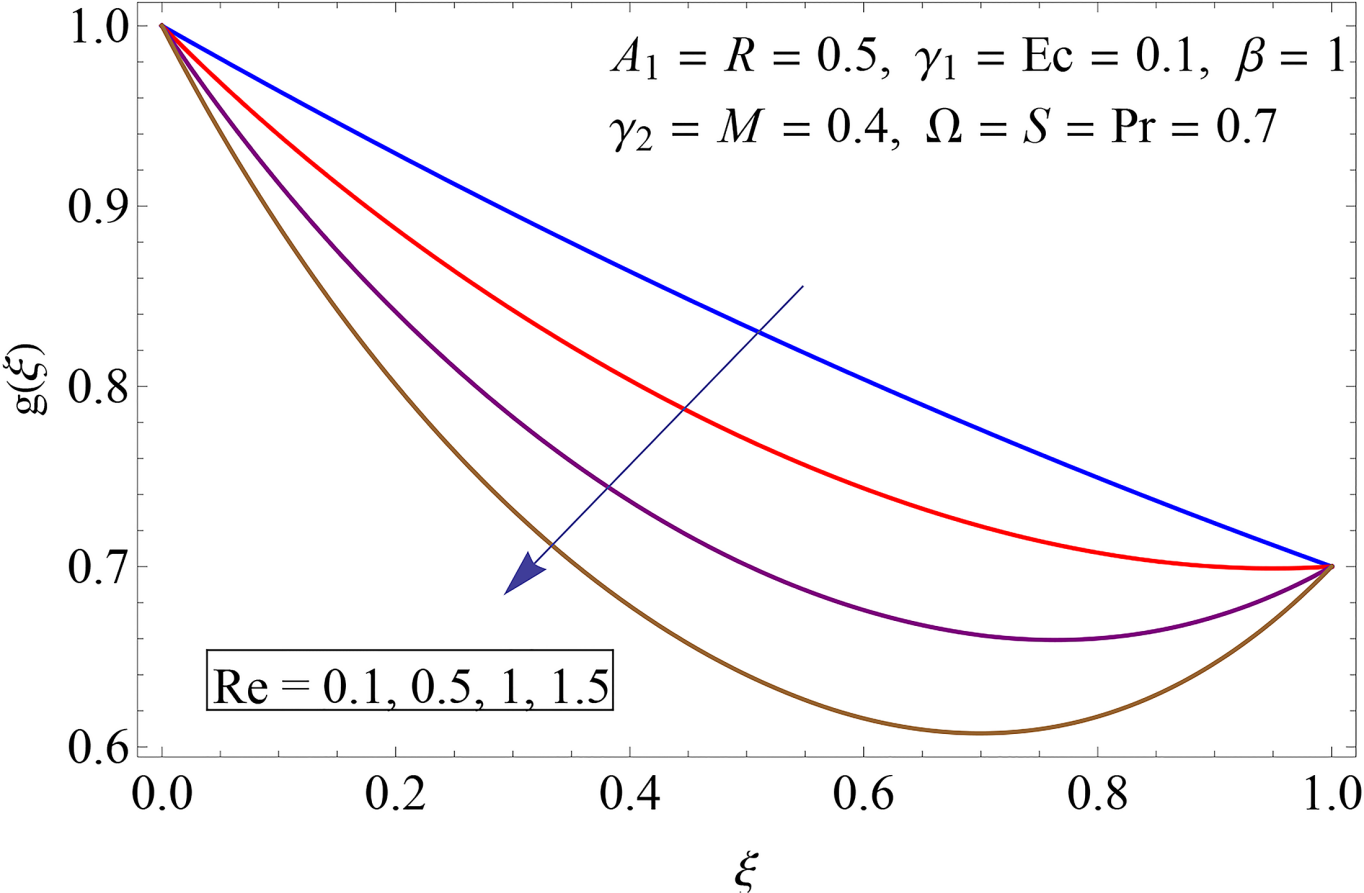
Impact of Re on *g*(*ξ*).

**Fig 10:**
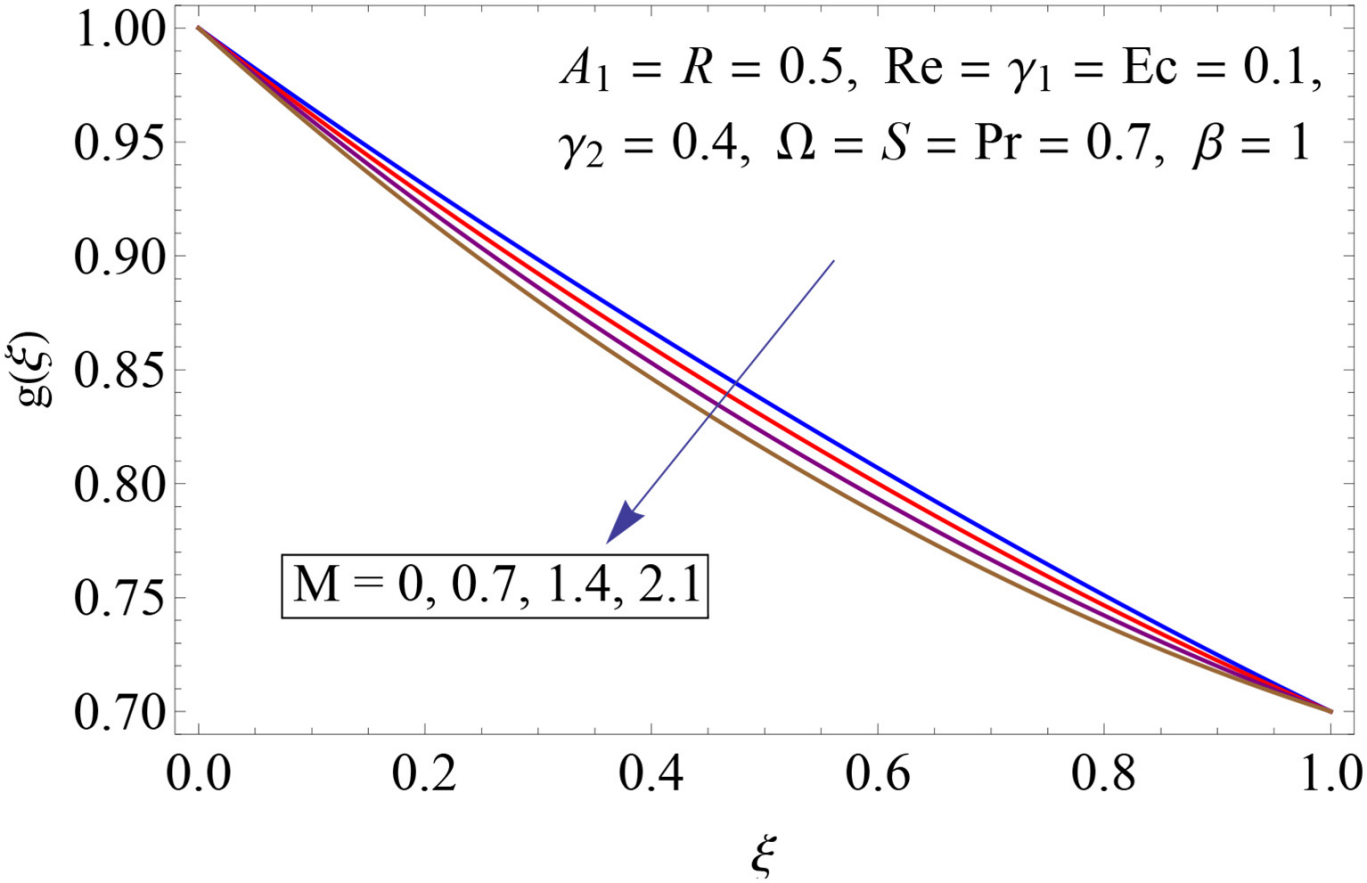
Impact of *M* on *g*(*ξ*).

**Fig 11:**
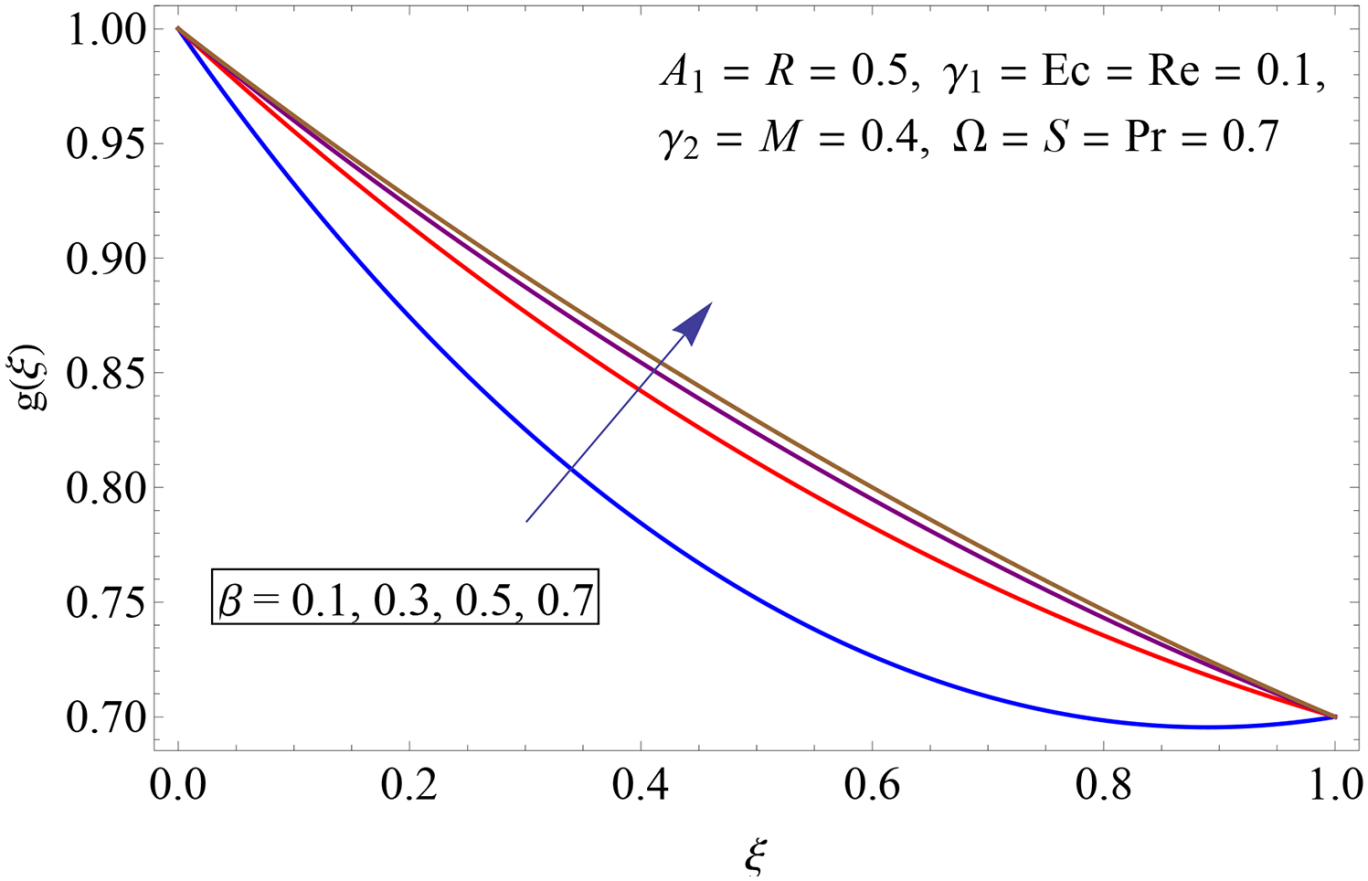
Impact of *β* on *g*(*ξ*).

**Fig 12:**
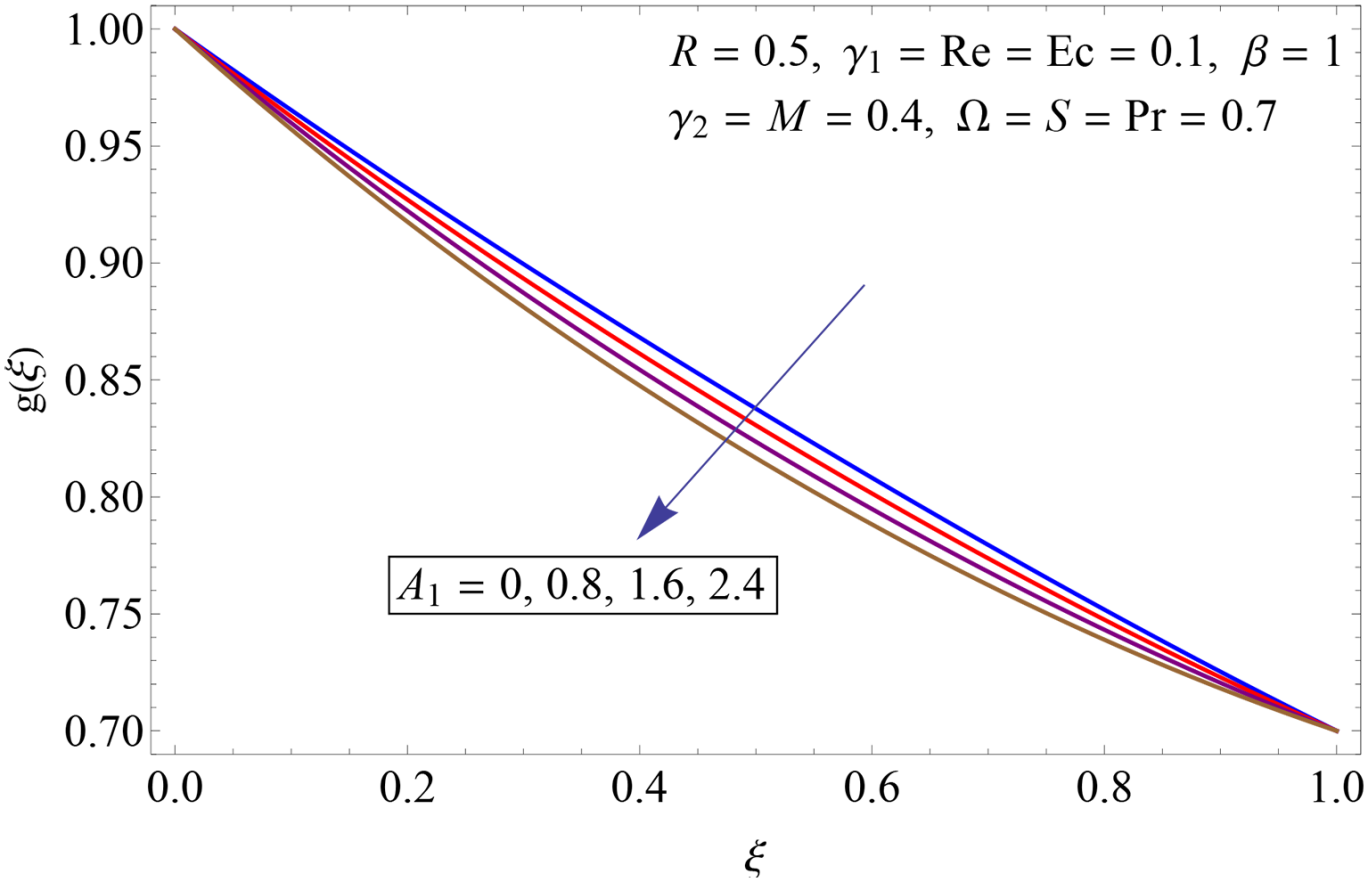
Impact of *A*_1_ on *g*(*ξ*).

**Fig 13:**
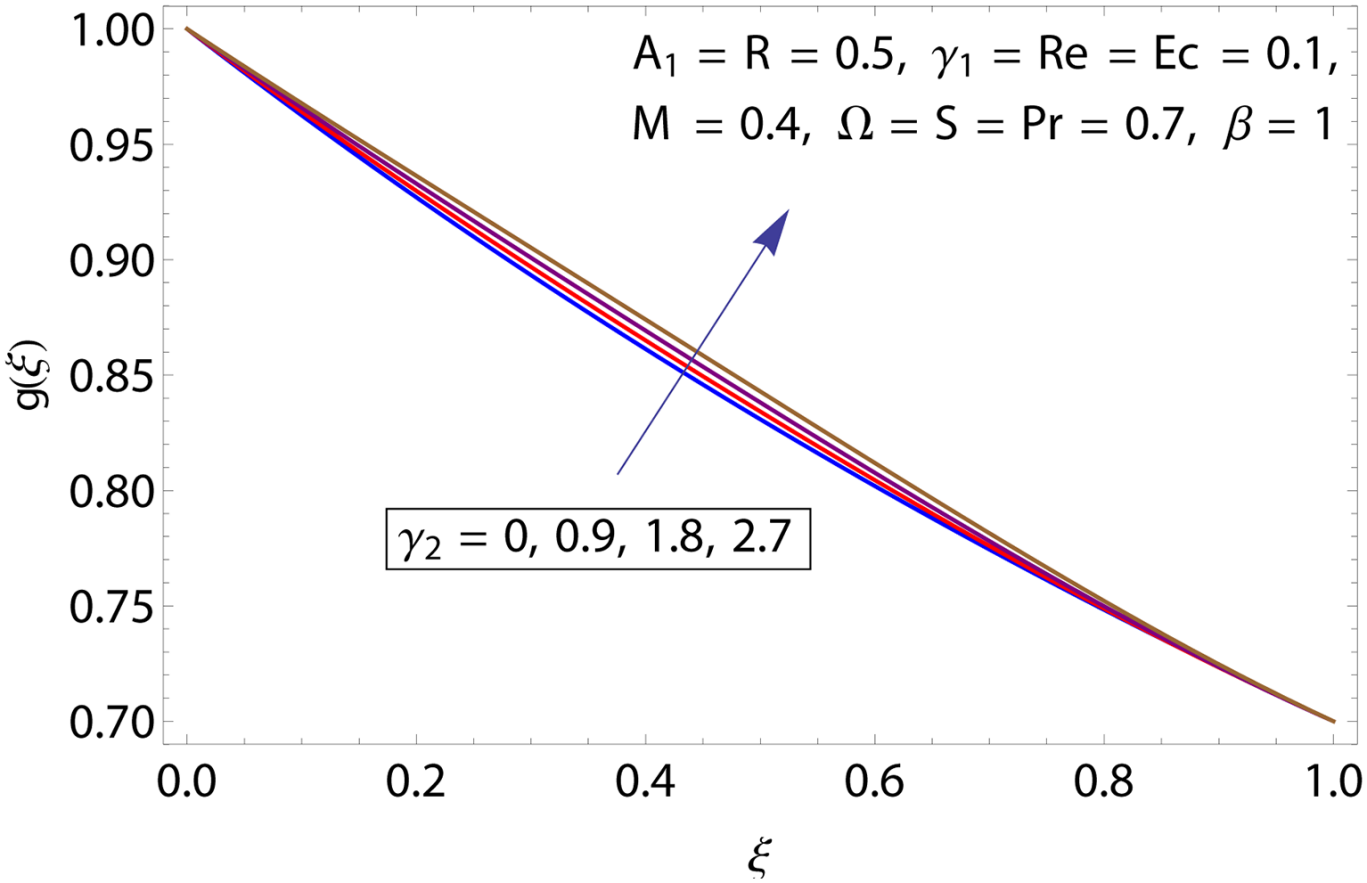
Impact of *γ*_2_ on *g*(*ξ*).

**Fig 14:**
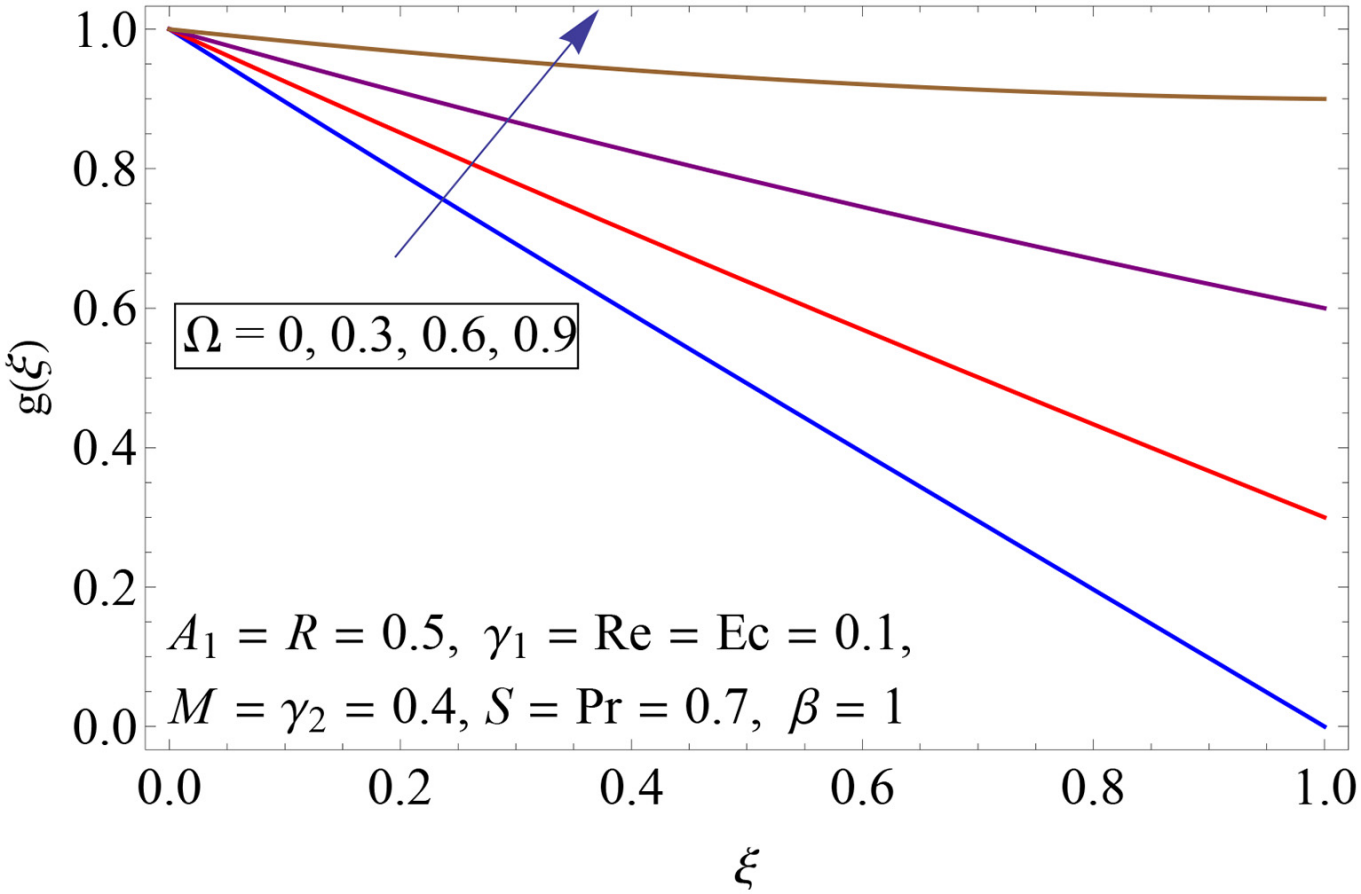
Impact of Ω on *g*(*ξ*).

Figs 9–14 show the impact of Reynolds number Re, Hartman number *M*, porosity parameter *β*, unsteadiness parameter *A*_1_, stretching parameter at upper disk *γ*_2_ and rotation parameter Ω on tangential velocity profile *g*(*ξ*). Fig 9 depicts the behavior of Reynolds number Re on tangential velocity profile *g*(*ξ*). For increasing values of Re tangential velocity *g*(*ξ*) decreases. Fig 10 shows the impact of Hartman number *M* on *g*(*ξ*). Tangential velocity decreases as we increase the values of *M* because magnetic field is a retarding force so when it applies to the fluid it slows down the motion of the fluid particles. Influence of porosity parameter *β* is portrayed in Fig 11. Larger values of *β* increase the permeability constant *K*_0_ and thus tangential velocity *g*(*ξ*) increases. Fig 12 shows the impact of *A*_1_ on tangential velocity. It is observed that for increasing values of *A*_1_ the tangential velocity decreases. Impact of stretching parameter of upper disk *γ*_2_ on tangential velocity *g*(*ξ*) is shown in Fig 13. Velocity increases due to the fact that for larger values of *γ*_2_ the stretching rate enhances. Fig 14 shows the behavior of rotation parameter Ω on tangential velocity *g*(*ξ*). Tangential velocity of fluid is increasing function of Ω.

### 5.3 Dimensionless temperature profile

**Fig 15:**
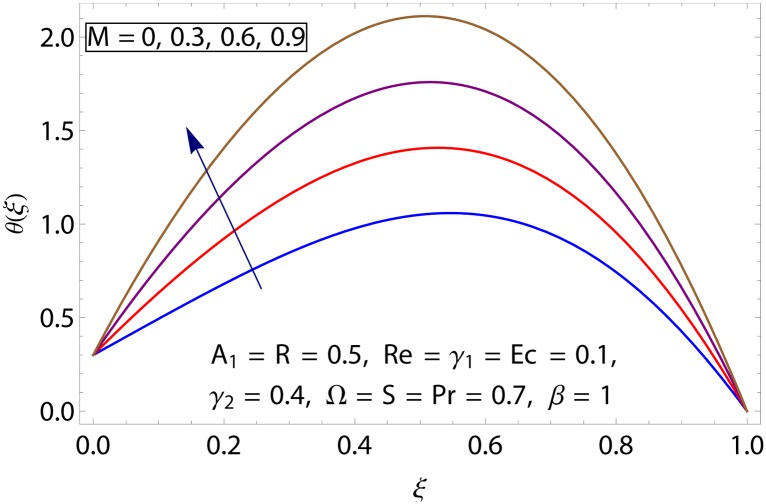
Impact of *M* on *θ*(*ξ*).

**Fig 16:**
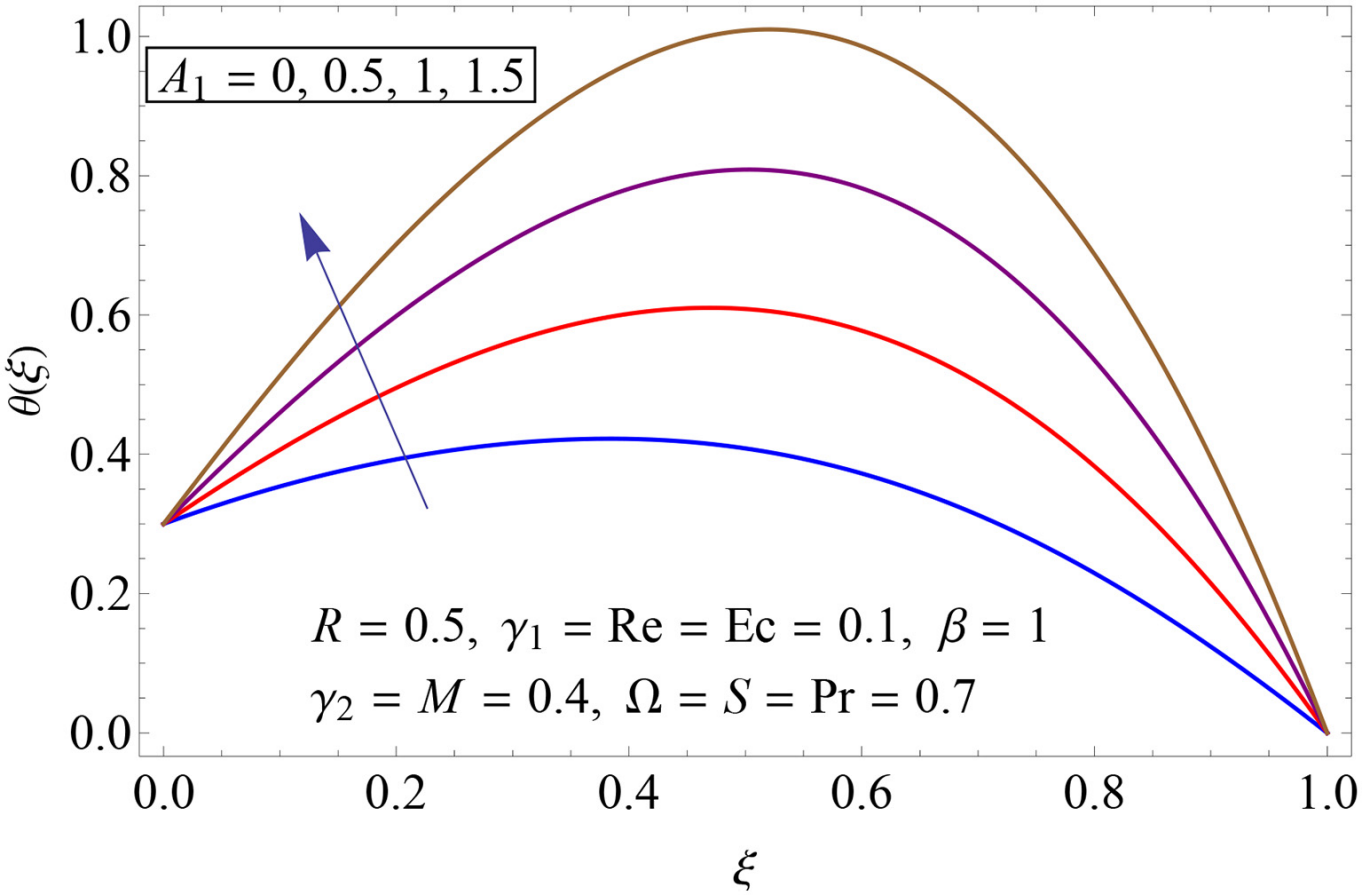
Impact of *A*_1_ on *θ*(*ξ*).

**Fig 17:**
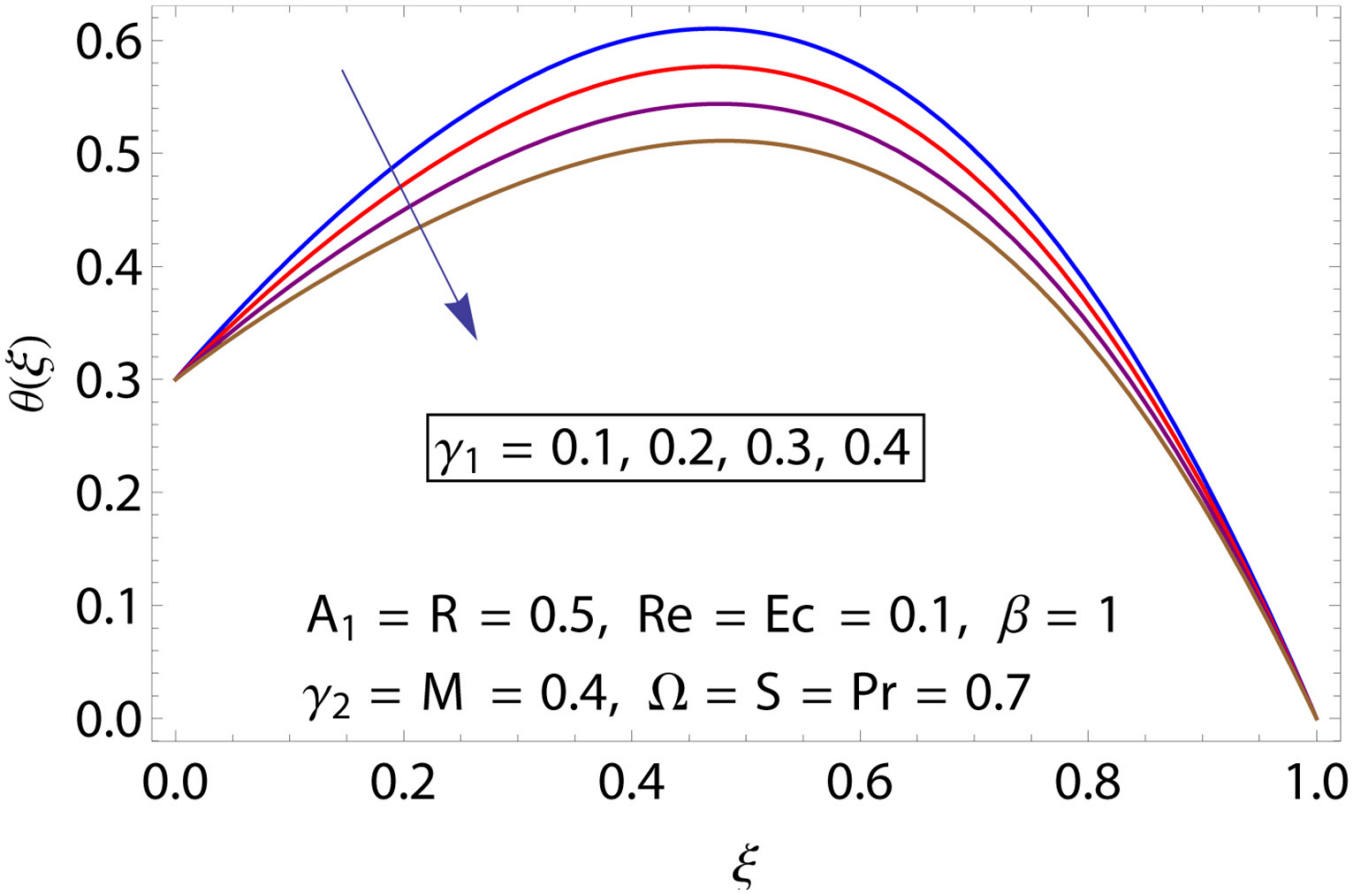
Impact of *γ*_1_ on *θ*(*ξ*).

**Fig 18:**
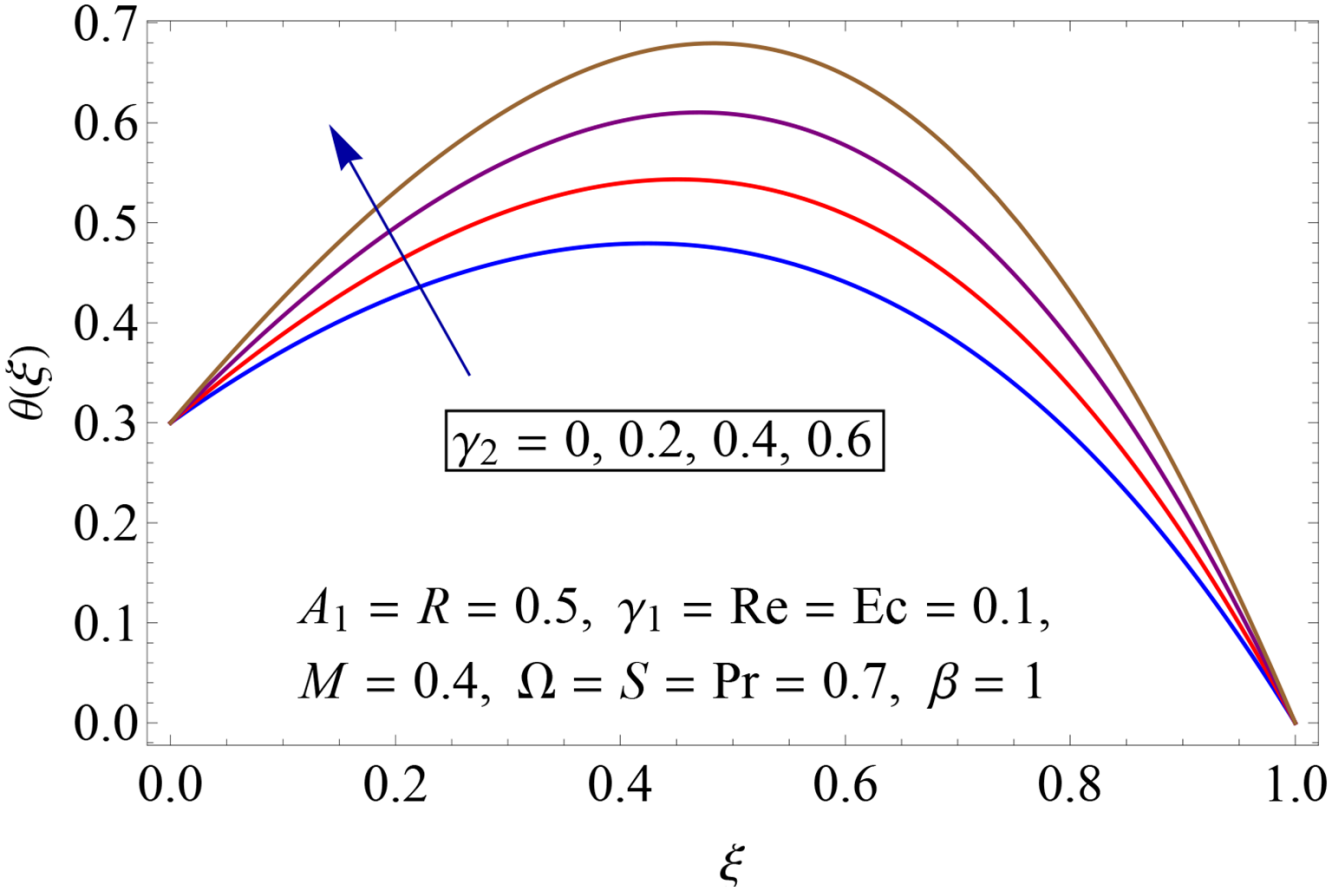
Impact of *γ*_2_ on *θ*(*ξ*).

**Fig 19:**
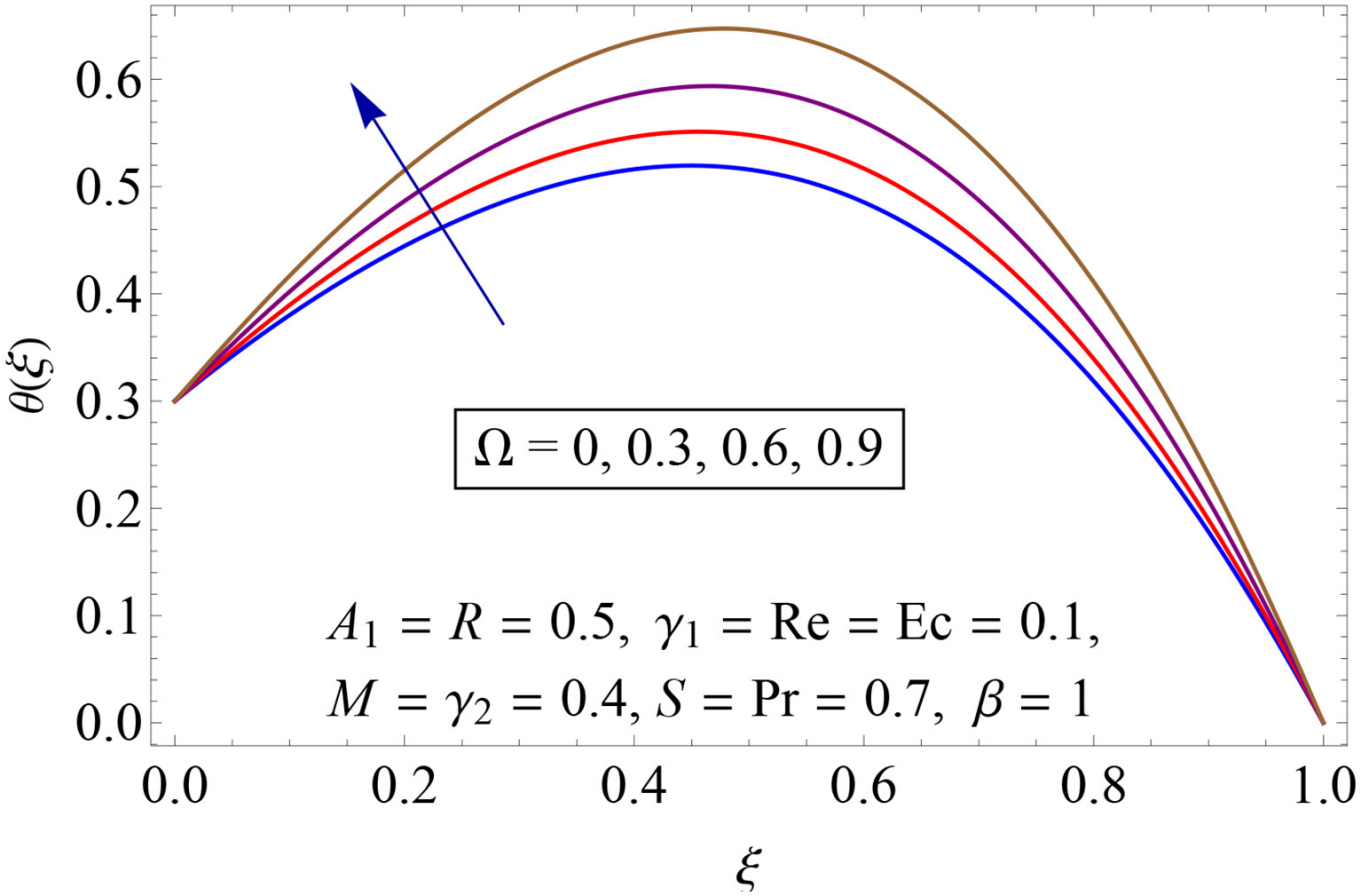
Impact of Ω on *θ*(*ξ*).

**Fig 20:**
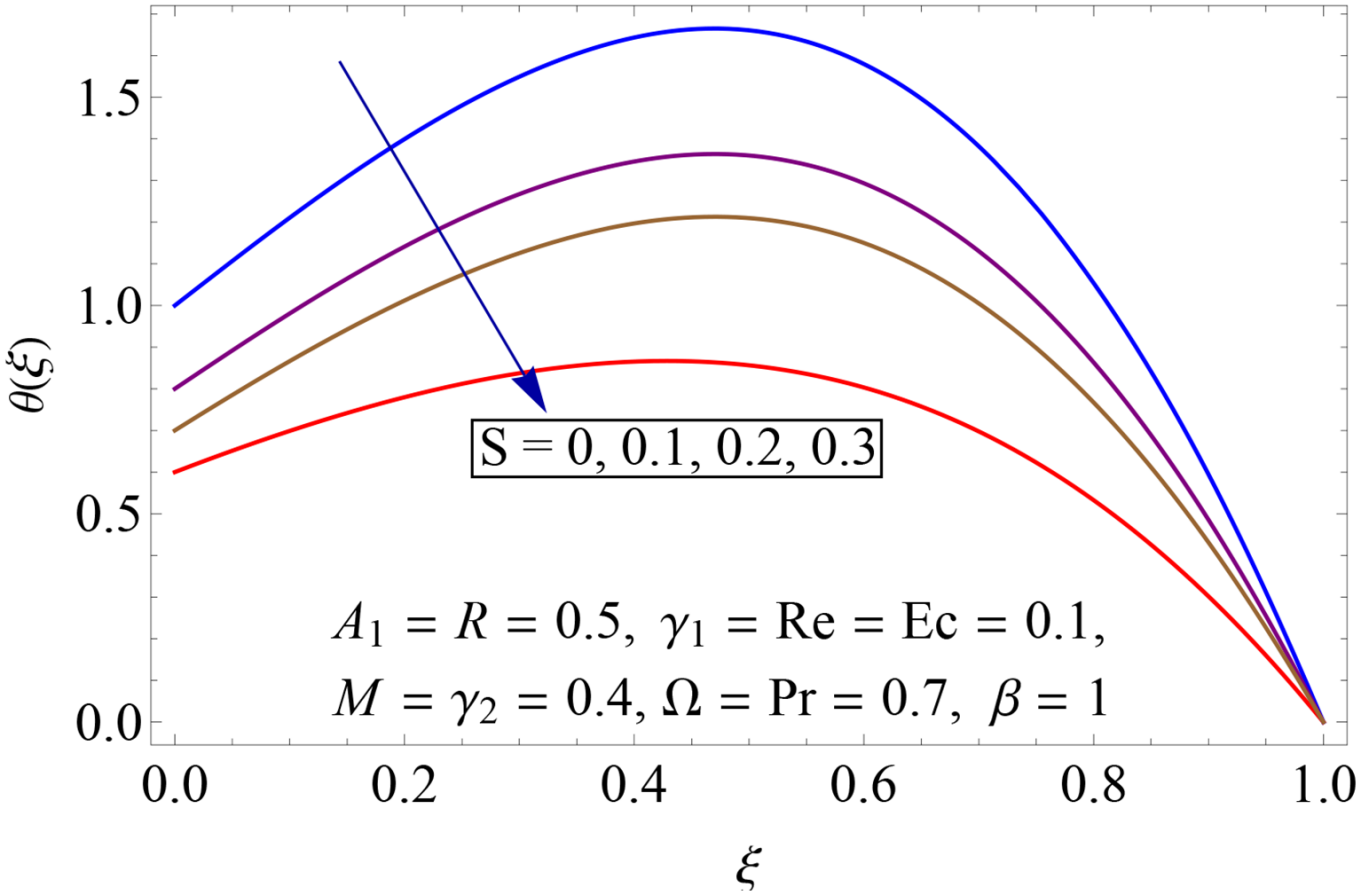
Impact of *S* on *θ*(*ξ*).

**Fig 21:**
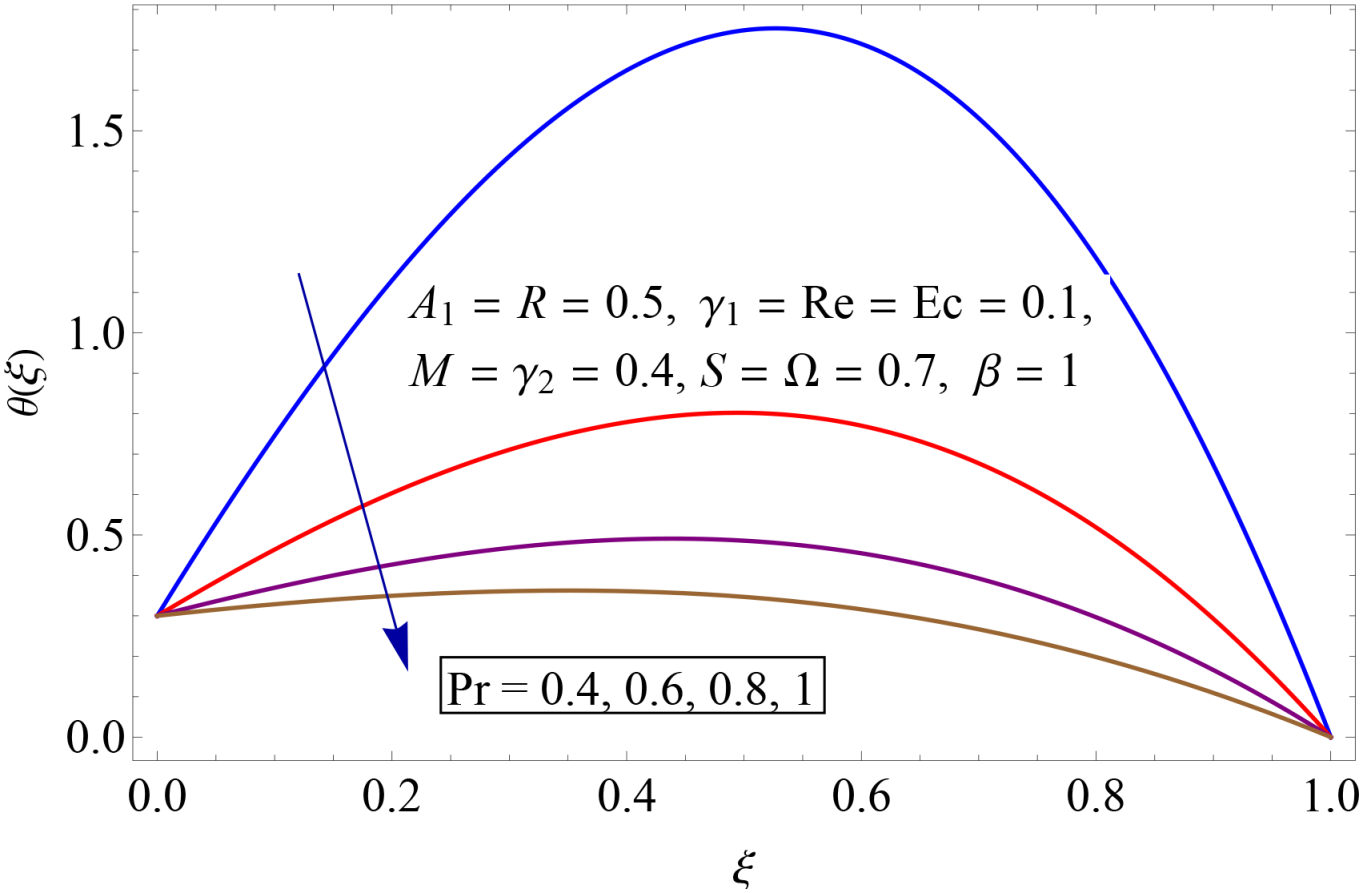
Impact of Pr on *θ*(*ξ*).

**Fig 22:**
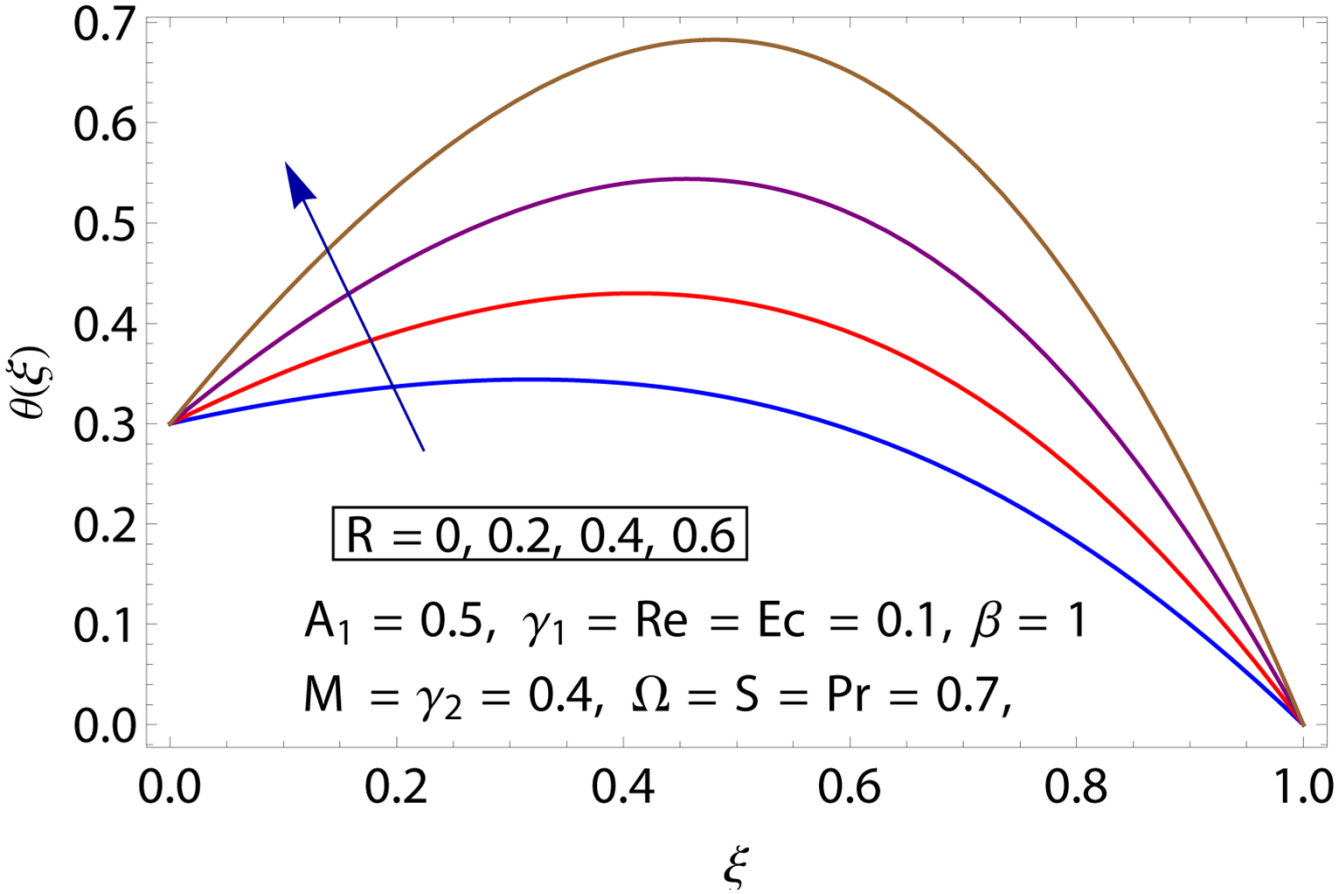
Impact of *R* on *θ*(*ξ*).

**Fig 23:**
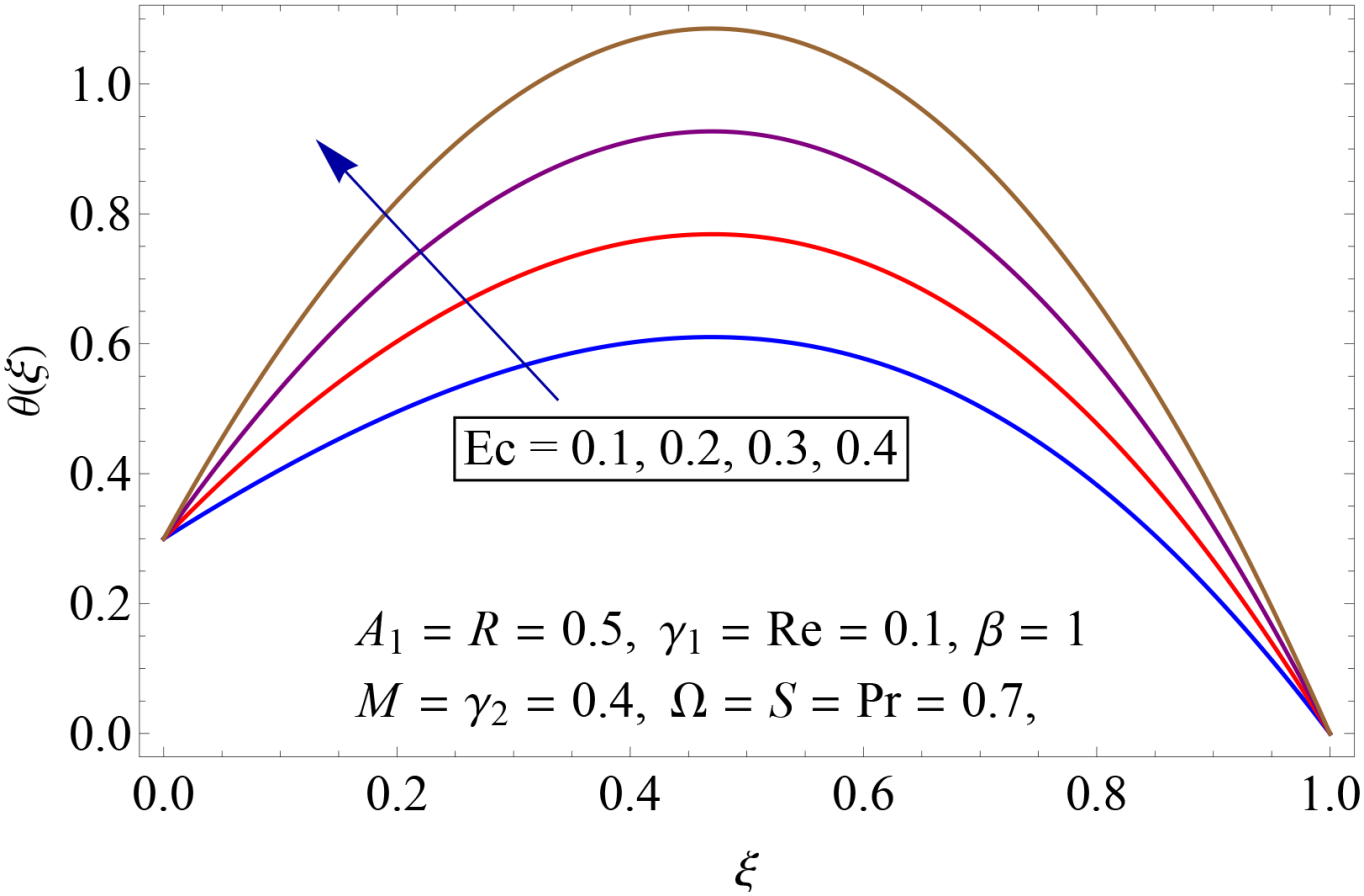
Impact of *Ec* on *θ*(*ξ*).

Influences of Hartman number *M*, unsteadiness parameter *A*_1,_ stretching parameters *γ*_1_ and *γ*_2,_ rotation parameter Ω, thermal stratification parameter *S*, Prandtl number Pr, radiation parameter *R* and Eckert number *Ec* on temperature profile *θ*(*ξ*) shown in the Figs 15–23. Fig 15 presents the influence of Hartman number *M* on temperature profile. Fluid temperature increases with larger values of Hartman number. In fact Lorentz force is also known as a resistive force which slows down the movement of particles. As a result heat is produced and temperature increases. Fig 16 portrays the effect of unsteadiness parameter *A*_1_ on fluid temperature. For increasing values of unsteadiness parameter the fluid temperature enhances. Figs 17 and 18 show the behavior of stretching parameters *γ*_1_ and *γ*_2_ on temperature profile *θ*(*ξ*). Influences of *γ*_1_ and *γ*_2_ on temperature profile are opposite. Increasing values of stretching parameter at lower disk *γ*_1_ reduce the temperature while temperature enhances by increasing stretching parameter of upper disk *γ*_2_ Impact of rotation parameter Ω on temperature profile is shown in Fig 19. For larger values of Ω. temperature of fluid enhances. Here more resistance is produced for increasing values of Ω and thus temperature increases. Fig 20 presents the behavior of temperature profile *θ*(*ξ*) for thermal stratification parameter *S*. Temperature profile and thermal boundary layer thickness are decrease via *S*. Actually the temperature difference between two disks decreases gradually which shows reduction in fluid temperature. Fig 21 depicts the behavior of Prandtl number Pr on *θ*(*ξ*). Temperature profile is decreasing function of Pr because thermal diffusivity decreases by increasing Prandtl number. Hence heat diffuses slowly and so temperature decreases. Fig 22 presents the behavior of temperature profile *θ*(*ξ*) for increasing values of radiation parameter *R*. Temperature profile is increasing function of *R*. As we increase the values of *R* mean absorption coefficient decreases. Thus rate of radiative heat transfer to the fluid increases. Fig 23 shows the influence of *Ec* on fluid temperature. It is noted that the temperature and thermal boundary layer thickness are enhances with increase in *Ec*. When *Ec* increases due to friction the heat produced in the fluid and thus temperature enhances.

### 5.4 Skin friction coefficient and Nusselt number

Influences of Reynolds number Re, Hartman number *M*, porosity parameter *β* and stretching parameters *γ*_1_ and *γ*_2_ on skin friction coefficients at the lower and upper disks are depicted in Table 2. Skin friction coefficient increases at lower disk for larger values of Re, *M*, *γ*_1_ and *γ*_2_ while it shows decreasing impact for increasing values of porosity parameter. Increasing values of Re, *M*, *β*, *γ*_1_ and *γ*_2_ enhance skin friction coefficient at upper disk. Table 3 shows the impact of thermal stratification parameter *S*, Prandtl number Pr, Reynolds number Re, Eckert number *Ec* and radiation parameter *R* on rate of heat transfer at the lower and upper disks. It is observed that impact of Pr, Re and *Ec* on Nusselt number at both disks is opposite. At lower disk Nusselt number is decreasing function of Pr, Re and *Ec* while it increases at upper disk. As we increase the values of *S* the heat transfer rate at both disks decreases. With increasing values of radiation parameter *R* the heat transfer rate at both disks enhances.

**Table 2:** Surface drag force at the lower and upper disks for different involved physical parameters.

Re	*M*	*β*	*γ*_1_	*γ*_2_	Re_*r*_ *C*_*f*1_	Re_*r*_ *C*_*f*2_
0.1	0.4	1	0.1	0.4	1.247797	1.833171
0.2					1.262808	1.844225
0.3					1.281529	1.857798
0.1	0.6				1.250470	1.833185
	0.8				1.253190	1.833234
	0.4	1.2			1.245606	1.833188
		1.4			1.244062	1.833215
		1.0	0.2		1.635499	2.033348
			0.3		2.028616	2.234147
			0.1	0.6	1.626163	2.636354
				0.8	2.008750	3.441569

**Table 3:** Heat transfer rate at the lower and upper disks for different involved physical parameters.

*S*	Pr	Re	*Ec*	*R*	−(1+*R*)*θ*′(0)	−(1+*R*)*θ*′(1)
0.2	0.7	0.1	0.1	0.5	1.195623	1.207451
0.4					0.8964356	0.9058187
0.6					0.5972487	0.6041857
0.7	0.8				0.4473201	0.4538518
	0.9				0.4469851	0.4543347
	0.7	0.2			0.4453776	0.4566894
		0.3			0.4431633	0.4599638
		0.1	0.3		0.4454042	0.4552091
			0.6		0.4420278	0.4579690
			0.1	0.7	0.5076551	0.5133683
				0.9	0.5676551	0.5733676

## 6. Concluding Remarks

Heat transfer and MHD flow analysis between two parallel rotating disks is carried out in presence of thermal radiation, stratification and Joule heating. Main findings of our problem are as follows:

- Magnitude of radial and axial velocities decreases near the lower disk for increasing values of Re.
- Opposite behavior of *γ*_1_ and *γ*_2_ at lower disk is observed for radial and axial velocities.
- Tangential velocity is decreasing function of Hartman number.
- Fluid temperature decays with increasing values of thermal stratification parameter and Prandtl number.
- Temperature profile enhances for larger values of Eckert number and radiation parameter.
- Surface drag force enhances at both disks for increasing values of Re, *M* and *γ*_1_.
- For larger values of Pr, Re and *Ec* heat transfer rate enhances.

## References

[1] Karman TV (1921) Uber laminare and turbulente Reibung. Zeit. Angew. Math. Mech. 1, 233–252.

[2] Cochran WG (1934) The flow due to a rotating disk. Proc. Camb. Philo. Soc. 30, 365–375

[3] Stewartson K (1953) On the flow between two rotating coaxial disks, Proc. Comb. Phil. Soc. 49, 333–341.

[4] Chapple PJ, Stokes VK (1962) On the flow between a rotating and a stationary disk. Report No. FLD 8. Dept. Mech. Eng. Princeton University.

[5] Mellor GJ, Chapple PJ, Stokes VK (1968) On the flow between a rotating and a stationary disk. J. Fluid Mech. 31, 95–112.

[6] Arora RC, Stokes VK (1972) On the heat transfer between two rotating disks. Int. J. Heat Mass Transf. 15, 2119–2132.

[7] Kumar SK, Tacher WI, Watson LT (1989) Magnetohydrodynamic flow between a solid rotating disk and a porous stationary disk. Appl. Math. Model. 13, 44–500.

[8] Yan WM, Soong CY (1997) Mixed convection flow and heat transfer between co-rotating porous disks with wall transpiration. Int. J. Heat Mass Transf. 40, 773–784.

[9] Soong CY, Wu CC, Liu TP, Liu TP (2003) Flow structure between two co-axial disks rotating independently,.Exp. Thermal Fluid Sci. 27, 295–311.

[10] Turkyilmazoglu M (2014) MHD fluid flow and heat transfer due to a shrinking rotating disk. Compt. Fluid 90, 51–56

[11] Gao ZK, Fang PC, Ding MS, Jin ND (2015) Multivariate weighted complex network analysis for characterizing nonlinear dynamic behavior in two-phase flow. Exp. Thermal Fluid Sci. 60, 157–164.

[12] Gao ZK, Yang YX, Zhai LS, Ding MS, Jin ND (2016) Characterizing slug to churn flow transition by using multivariate pseudo Wigner distribution and multivariate multiscale entropy. Chem. Eng. J. 291, 74–81.

[13] Gao ZK, Yang Y, Zhai L, Jin N, Chen G (2016) A four-sector conductance method for measuring and characterizing low-velocity oil-water two-phase flows. IEEE Trans. Instrumentation Measurement 10.1109/TIM.2016.2540862 (in press).

[14] Gao ZK, Yang YX, Fang PC, Jin N, Xia CY, Hu LD (2015) Multi-frequency complex network from time series for uncovering oil-water flow structure, Scientific Reports 5, 8222 10.1038/srep08222 25649900 PMC4316157

[15] Zhang Y, Lu T, Jiang PX, Zhu YH, Wu J, Liu CL (2016) Investigation on thermal stratification and turbulent penetration in a pressurizer surge line with an overall out-surge flow. Annals Nuclear Energy 90, 212–233.

[16] Mukhopadhyay S (2013) MHD boundary layer flow and heat transfer over an exponentially stretching sheet embedded in a thermally stratified medium. Alex. Eng. J. 52, 259–265.

[17] Hayat T, Imtiaz M, Alsaedi A (2016) Unsteady flow of nanofluid with double stratification and magnetohydrodynamics. Int. J. Heat Mass Tranf. 92, 100–109.

[18] Hayat T, Hussain T, Shahzad SA, Alsaedi A (2014) Thermal and concentration stratifications effects in radiative flow of Jeffrey fluid over a stretching sheet. Plos One 9, (10), e107858. 25275441 10.1371/journal.pone.0107858 PMC4183484

[19] Srinivasacharya D, Upendar M (2013) Effect of double stratification on MHD free convection in a micropolar fluid. J. Egypt. Math. Soci. 21, 370–378.

[20] Hayat T, Imtiaz M, Alsaedi A (2015) MHD 3D flow of nanofluid in presence of convective conditions. J. Mol. Liq. 212, 203–208.

[21] Sheikholeslami M, Bandpy MG, Ganji DD, Soleimani S (2013) Effect of a magnetic field on natural convection in an inclined half-annulus enclosure filled with Cu—water nanofluid using CVFEM. Adv. Powder Tech. 24, 980–991.

[22] Hsiao KL (2016) Stagnation electrical MHD nanofluid mixed convection with slip boundary on a stretching sheet. Appl. Therm. Eng. 98, 850–861.

[23] Zhang C, Zheng L, Zhang X Chen G (2015) MHD flow and radiation heat transfer of nanofluids in porous media with variable surface heat flux and chemical reaction. Appl. Math. Modeling, 39, 165–181.

[24] Ahmad R, Mustafa M, Hayat T, Alsaedi A (2016) Numerical study of MHD nanofluid flow and heat transfer past a bidirectional exponentially stretching sheet. J. Magn. Magn. Mater. 407, 69–74.

[25] Rashidi S, Dehghan M, Ellahi R, Riaz M, Abad MTJ (2015) Study of stream wise transverse magnetic fluid flow with heat transfer around a porous obstacle. J. Magn. Magn. Mat. 378, 128–137.

[26] Sheikholeslami M, Ellahi R (2015) Simulation of ferrofluid flow for magnetic drug targeting using Lattice Boltzmann method. J. Zeitschrift Fur Naturforschung A. 70, (2), 115–124.

[27] Hsiao KL (2011) MHD mixed convection for viscoelastic fluid past a porous wedge. Int. J. Non-Linear Mech. 46, 1–8.

[28] Hsiao KL (2015) Corrigendum to Heat and mass mixed convection for MHD viscoelastic fluid past a stretching sheet with Ohmic dissipation. Commu. Nonlinear Sci. Numer. Simulate 28, 232.

[29] Ganji DD, Malvandi A (2014) Natural convection of nanofluids inside a vertical enclosure in the presence of a uniform magnetic field. Powder Tech. 263, 50–57.

[30] Moniem AA, Hassanin WS (2013) Solution of MHD flow past a vertical porous plate through a porous medium under oscillatory suction. Sci. Research 4, 694–702.

[31] Ellahi R, Bhatti MM, Riaz A, Sheikholeslami M (2014) Effects of Magnetohydrodynamics on Peristaltic flow of Jeffrey fluid in a rectangular duct through a porous medium, J. Porous Media 17, (2), 143–157.

[32] Hayat T, Imtiaz M, Alsaedi A, Mansoor R (2015) Magnetohydrodynamic three-dimensional flow of nanofluid by a porous shrinking surface. J. Aerospace Eng. 29, (2), 10.1061/(ASCE)AS.1943-5525.0000533

[33] Yang L, Shen H (2015) Effects of the porous media distribution on the performance improvement for isothermal chamber. Appl. Therm. Eng. 86, 301–308.

[34] Abad MTJ, Saedodin S, Aminy M (2016) Heat transfer in concentrated solar air-heaters filled with a porous medium with radiation effects: A perturbation solution. Renew. Energy 91, 147–154.

[35] Hatami M, Sheikholeslami M, Ganji DD (2014) Nanofluid flow and heat transfer in an asymmetric porous channel with expanding or contracting wall. J. Mol. Liq. 195, 230–239.

[36] Zhao Y, Tang GH (2016) Monte Carlo study on extinction coefficient of silicon carbide porous media used for solar receiver. Int. J. Heat Mass Transf. 92, 1061–1065.

[37] Bhattacharyya K, Mukhopadhyay S, Layek GC, Pop I (2012) Effects of thermal radiation on micropolar fluid flow and heat transfer over a porous shrinking sheet. Int. J. Heat Mass Transf. 55, 2945–2952.

[38] Sheikholeslami M, Ashorynejad HR, Ganji DD, Rashidi MM (2014) Heat and mass transfer of a micropolar fluid in a porous channel. Commun.Numer. Analysis 2014.

[39] Hayat T, Qayyum S, Imtiaz M, Alsaedi A (2016) Impact of Cattaneo-Christov heat flux in Jeffrey fluid flow with homogeneous-heterogeneous reactions. Plos One 11, (2), e0148662 10.1371/journal.pone.0148662 26859675 PMC4747563

[40] Shahzad SA, Abbasi FM, Hayat T, Alsaadi FE (2015) Model and comparative study for peristaltic transport of water based nanofluids. J. Mol. Liq. 209, 723–728.

[41] Hayat T, Qayyum S, Imtiaz M, Alsaedi A (2016) Three-dimensional rotating flow of Jeffrey fluid for Cattaneo-Christov heat flux model. AIP Advances 025012.

[42] Lin Y, Zheng L, Chen G (2015) Unsteady flow and heat transfer of pseudoplastic nano liquid in a finite thin film on a stretching surface with variable thermal conductivity and viscous dissipation. Powder Tech. 274, 324–332.

